# A Dynamic Dual Process Model for Binary Choices: Serial Versus Parallel Architecture

**DOI:** 10.1007/s42113-023-00186-1

**Published:** 2023-12-06

**Authors:** Adele Diederich

**Affiliations:** Department of Psychology, Carl von Ossietzky University, Oldenburg, Germany

**Keywords:** Dual process models, Two-stage stochastic process, Sequential sampling, Serial processing, Parallel processing

## Abstract

Dual process theories have become increasingly popular in psychology, behavioral economics, and neuroscience, assuming that two processes, here generically labeled as System 1 and System 2, have antagonistic characteristics such as automatic versus deliberate, impulsive versus rational, fast versus slow, and more. In decision-making a choice results from an interplay of these two systems. However, most existent dual-process approaches are merely verbal descriptions without providing the means of rigorous testing. The prescribed dynamic dual process model framework is based on stochastic processes and produces testable qualitative and quantitative predictions. In particular, it makes precise predictions regarding choice probability, response time distributions, and the interrelation between these quantities. The focus of the present paper is on the architecture of the two postulated systems: serial versus parallel processing. Using simulation studies, I illustrate how different factors (timing of System 1, time constraint, and architecture) influence model predictions for binary choice situations. The serial and 6 parallel processing versions of the framework are fitted to published data.

## Introduction

The idea of two separate systems originates in philosophy and is most prominently expressed in dualism (Robinson, 2020). Related to the basic concept and their modifications (Fiala et al., 2012), the notion of dual processes and dual systems received increased attention in psychology, and more recently, in neuroscience and related fields. Dual-process/system approaches describe cognitive performances as a product of two interacting systems (Gawronski & Creighton, 2013; Evans, 2008; Kahneman, 2011). The two processes/systems have been named, for example, as symbolic vs. associative in reasoning (Sloman, 1996), heuristic vs. analytic in reasoning (Evans, 2006), associative vs. rule-based in memory (Smith & DeCoster, 2000), affective vs. deliberate in decision-making (Loewenstein et al., 2015; Stanovich & West, 2000), intuitive vs. reflective in moral judgment (Gürçay and Baron, 2017), or simply termed System 1 and System 2 (Kahneman & Frederick, 2002; Kahneman, 2011). For overviews and nomenclature, see, e.g., (Evans, 2008; Milli et al., 2021; DeNeys, 2021).^1^ System 1 is assumed to lead to biased decisions revealed in preference shifts or preference reversals.

Neuroscience research also suggests that the human mind is composed of multiple systems that approach decisions in distinct ways: a fast, habit-based system and a slow, goal-directed system for value-based decision-making (e.g., Dolan and Dayan, 2013, forareview). DeMartino and colleagues (De Martino et al., 2006), in the context of risky decision-making and framing, linked the two systems to activation in the brain: Bilateral amygdala (Amyg) activation and anterior cingulate cortex (ACC) activation. They link activities in the respective areas to risk attitudes in a gain frame and a loss frame (Kahneman & Tversky, 1979; Tversky & Kahneman, 1981).

Dual process models have yet to be applied to perceptual tasks to a far less extent. Here the processes have been described as automatic vs. controlled in detection, search, attention, and perceptual learning (Schneider & Shiffrin, 1977; Shiffrin & Schneider, 1977), ambient vs. focused in environmental perception (Morin et al., 2007), intentional vs. stimulus-driven for congruent and incongruent trials in visual perception (Devaine et al., 2014). Biases in this context are manifested in the proportion of correct and incorrect responses.

The two systems are often assumed to operate in an antagonistic manner, for instance, intuition versus rationality and rapidness versus slowness.

Despite the increasing number of approaches postulating two (or more) separate systems, there are hardly any formal mathematical models that can account for the dynamics of the interaction between the two systems and allow for precise predictions. Most dual process models are simply verbal descriptions of the two systems without generating testable quantitative predictions (Diederich and Trueblood, 2018). The attribution of one system or the other to an observation occurs post hoc and often ad hoc. Furthermore, there are debates over how the two systems interact. Most theories assume that the two systems occur sequentially; the slower System 2 modifies the response of the fast System 1. A few other approaches favor competitive-parallel processing (see, e.g., DeNeys, 2021; Evans & Stanovich, 2013; Keren & Schul, 2009; Milli et al., 2021, for discussion and criticism). None of them provides a mechanism for testing the different architectures.^2^ Formalizing the dual process assumptions to allow for predictions and rigorous testing would solve some of the issues that proposers and opponents of this approach hold.

There are few cases where formal models have been proposed, for instance, for self-control (Brocas & Carrillo, 2014; Fudenberg & Levine, 2006); for probabilistic reasoning (Klauer et al., 2010); for risky decision-making (Mukherjee, 2010; Loewenstein et al., 2015); for intertemporal choice (Fudenberg & Levine, 2006; Loewenstein et al., 2015). However, most of these models average across the two postulated systems, for instance, by combining the two values independently attributed to System 1 and 2, making the notion of two systems questionable.

More importantly, these models are silent regarding the timing of the two systems, which is nonetheless a core feature of verbal models on dual process theories. For instance, because System 1 is assumed to be fast, faster choices are associated with intuition. Likewise, because System 2 is assumed to be slow, slower responses are assumed to indicate rationality. However, as Krajbich et al. (2015) showed in detail, such reverse inference is problematic because it does not consider other sources of variability in the data that can influence choice speed (e.g., choice difficulty). Furthermore, even when a processing architecture is mentioned, none of them incorporates them into the model.

In the following, I describe a dynamic stochastic modeling framework of dual process theory, which provides an account both of the timing and interplay of the two systems. The framework is based on research using multistage sequential sampling models (Diederich, 2016) and was applied to risky choices presented in gain and loss frames (Diederich and Trueblood, 2018) and intertemporal choice situations (Diederich & Zhao, 2019) both assuming serial processing. Here, I extend the model to parallel processing and compare it to predictions of serial processing. For ease of communication, the model outline and predictions are shown for decision-making situations with binary choice options.

The remainder of this article is structured as follows. The “Dynamic-Stochastic Dual Process Framework: Basic Assumptions” section introduces basic assumptions of the underlying stochastic process and provides examples for valence constructions in System 1 and System 2 for preferential choices. The “Dynamic-Stochastic Dual Process Framework: Architechtures” section discusses the dynamic-stochastic dual process with two different processing structures (serial and pseudo-parallel) and provides qualitative and quantitative predictions shown in the context of framed risky choices. The paper ends with concluding remarks.

## Dynamic-Stochastic Dual Process Framework: Basic Assumptions

The model framework as presented here applies to binary choice situations in general, for instance, to perceptual decision-making, (e.g., Diederich, 2008, 2016; Diederich & Busemeyer, 2006) or preferential choice with two multiattribute choice options (e.g., Diederich, 1997, 2003; Diederich & Busemeyer, 1999). The choice alternatives are labeled *A* and *B*.

### Process Assumptions

For perceptual decision-making, decisions are based on an *information state*. Information in this context usually means any changes in the central nervous system that translate perception and cognition into action (Luce, 1986; Smith, 2000). For preferential decision-making, decisions are based on a *preference state* (Busemeyer & Townsend, 1993; Diederich, 1997). Here it is called *evidence state*, denoted by *X*(*t*), to cover both contexts, presenting the relative evidence strength for choosing one option (*A*) over another option (*B*) at time *t*.

Evidence strength is updated from one moment, *t*, to the next, $t+h$, by an input valence, *V*(*t*), reflecting the momentary comparison of consequences produced by imagining the choice of either option *A* or *B* with

1 $$\begin{aligned} V(t)=V^A(t) -V^B(t), \end{aligned}$$

where $V^A(t)$ is the valence for option *A* at time *t* and $V^B(t)$ the one for option *B*. The valence fluctuates because the decision maker’s attention switches back and forth to the respective anticipated consequences.

For dual processes, the input valence also depends on the system *i* in which the decision maker (DM) is operating, where $i = 1, 2$. The evidence process can be described by the following equation

2 $$\begin{aligned} X(t+h) = X(t) + {{V}}_i(t+h), \end{aligned}$$

where *h* is a small time unit and $V_i(t)$ is the system-specific valence input. Note that the input valence ${{V}}_i(t+h)$ changes during the deliberation time when operation in one system switches to operation in another system. Thus the entire process is non-time homogeneous which makes it unique among sequential sampling approaches. A formal derivation in provided in the “Digression: More than Two Sub-processes” section. A positive value of *X*(*t*) represents a momentary evidence in the direction of choosing option *A* whereas a negative value of *X*(*t*) represents a momentary evidence in the direction of choosing option *B*. The mean valence when comparing the choice options and operating within system *i*, is $E[V_i(t)] = \mu _i t$. The parameter $\mu _i$, called the *drift rate*, indicates the direction and strength of evidence towards choosing option *A* or *B* while operating in system *i*.^3^ In particular, for $\mu _i >0$ evidence is directed towards choosing option *A* whereas for $\mu _i <0$ it is directed towards choosing *B*. The larger the absolute value of $\mu _i$, the stronger is the evidence for the given choice option. For $\mu _i = 0$, the DM is indifferent between the two choice options. In the context of perceptual choices, $\mu _i = 0$ relates to guessing. The preference process stops and a final decision is made as soon as the preference state exceeds a decision threshold or boundary: If $X(t) > \theta _A$, the decision maker (DM) chooses option *A* and if $X < - \theta _B$, the DM chooses option *B*. The decision threshold is assumed to be set prior to the decision task by the DM and depends, among other things, on the time available for a decision. Under short time limits, the boundaries are assumed to be smaller than under no time limits.Thus, the probability of choosing option *A* over *B* is determined by *X*(*t*) reaching the positive threshold before reaching the negative threshold.

### Valence Construction

The dynamic dual process framework assumes that evidence strength is updated according to Eq. (1), that is, valences in System 1 are $V_1(t)= V_1^A(t) -V_1^B(t)$ with drift rate $\mu _1$. Similar, the valences in System 2 are $V_2(t)= V_2^A(t) -V_2^B(t)$ with drift rate $\mu _2$. The valence is subject to further modeling. Its defining features depend on the research topic and on the modeler’s assumptions about the underlying processes, expressed in various functional forms. Examples are shown in the “Examples” section.

However, regardless of the specific functional form, eventually, the resulting value is mapped onto the mean valence (drift rate). Thus, according to the notation introduced earlier, the drift rate when operating in System 1 indicates

3 $$\begin{aligned} \mu _1 {\left\{ \begin{array}{ll}{>0} &{} \text {in favor of choosing { A} } \\ = 0 &{} \text {indifferent between choosing { A} and { B}}\\ < 0 &{} \text {in favor of choosing { B} } \end{array}\right. } \end{aligned}$$

The same holds for System 2:

4 $$\begin{aligned} \mu _2 {\left\{ \begin{array}{ll}>0 &{} \text {in favor of choosing { A} } \\ {=0} &{} \text {indifferent between choosing { A} and { B} }\\ < 0 &{} \text {in favor of choosing { B}} \end{array}\right. } \end{aligned}$$

### Examples

The following shows examples of valence constructions for preferential choices. Descriptive (psychological) choice models have been associated with System 1 and involved in decision biases (see Kahneman, 2011, for an overview). Normative (rational) choice models have been linked to System 2. For perceptual decision-making, the processes are related to stimulus properties including temporal and spatial arrangements, which is not discussed here further.

#### Example 1

In the context of risky choices Loewenstein et al. (2015) described preferences in System 1 by a motivational function *M*(*R*, *a*), which incorporates a variable *a* that captures the intensity of affective motivations (p. 59 Loewenstein et al., 2015), and is weighted by a function $h(W,\sigma )$ that reflects the willpower strength *W* and cognitive demands $\sigma$. Specifically,

$$\begin{aligned} M(x,a) = \sum _i w(p_i)v(x_i,a), \end{aligned}$$

where *w*(*p*) is a probability-weighting function, and *v*(*x*, *a*) is a value function that incorporates loss aversion and specified as

5 $$\begin{aligned} v(x,a) = {\left\{ \begin{array}{ll}{a\,u(x)} &{} \text {if } x \ge 0 \\ a \lambda \, u(x) &{} \text {if } x < 0 \end{array}\right. } \end{aligned}$$

with $\lambda > 0$ reflecting the degree of loss aversion, and

6 $$\begin{aligned} w(p)= c + bp \end{aligned}$$

with $w(0)=0$, $w(1) = 1$, and $0< c < 1-b$. The form of the function $h(W,\sigma )$ is not specified in (Loewenstein et al., 2015) but meant to be decreasing in *W* and increasing in $\sigma$.

Given a simple gamble with specific values $x_A$ and $x_B$ for option *A* and *B*, respectively, and their respective probabilities $p_A$ and $p_B$ to win/loss them, the drift rate in System 1 is determined by

7 $$\begin{aligned} \mu _1 =h(W,\sigma ) \cdot [ w(p_A)\cdot v(x_A,a) - w(p_B)\cdot v(x_B,a)]. \end{aligned}$$

For System 2 preferences were constructed according to expected utility theory (see Loewenstein et al., 2015, for details) leading to a drift rate

8 $$\begin{aligned} \mu _2 = p_A\cdot u(x_A) - p_B\cdot u(x_B). \end{aligned}$$

Note that Loewenstein et al. (2015) assumed that both processes operate simultaneously as a one-stage model expressed by the sum of both processes. That is, the subjective value of an option is

9 $$\begin{aligned} {\textbf {V}}(x) = \sum _i p_i u(x_i) + h(W, \sigma ) \cdot M(x,a). \end{aligned}$$

Furthermore, the model static and deterministic but, as shown here, can be converted into a dynamic-stochastic model.

#### Example 2

In the context of risky choices presented in a gain and loss frame Diederich and Trueblood (2018) assumed that preferences in System 1 are constructed according to prospect theory (PT, Kahneman & Tversky, 1979) and preferences in System 2 are consistent with expected utility theory. As in the previous examples, both models are static and deterministic but hypotheses derived from them can be implemented in the dynamic-stochastic framework.

According to prospect theory, the DM is risk avoiding in the gain domain (positive frame, e.g., winning money, saving people) and risk-seeking in the loss domain (negative frame, e.g. losing money, people die) ( (Kahneman & Tversky, 1979; Tversky & Kahneman, 1992). Assume that option *A* is a sure option (probability for the outcome is 1) and option B is a gamble. In a gain-framed scenario, the DM is expected to choose the sure option *A* over the gamble *B* and in a loss-framed scenario, the DM is expected to choose the gamble *B* over the sure option *A*.

Thus, according to the notation introduced earlier ($\mu >0$ favors choosing *A*, $\mu <0$ favors choosing *B*) the drift rate for choosing when operating in System 1 is

10 $$\begin{aligned} \mu _1 {\left\{ \begin{array}{ll}{>0} &{} \text {in a gain frame } \\ < 0 &{} \text {in a loss frame } \end{array}\right. } \end{aligned}$$

For instance, without going into any mathematical specifications of the functions defining PT (see, e.g., Fox & Poldrack, FoxPoldrack09, for various examples) the value function in the gain domain is concave and the one in the loss domain convex, the latter steeper than the former (Kahneman & Tversky, 1979; Tversky & Kahneman, 1992). Thus we can assume that the absolute value of $\mu _1$ is larger in the loss frame than in the gain frame. The risk attitude according to expected utility theory (vonNeumann & Morgenstern, 1953) does not change across domains (gain or loss). Either a DM is risk-neutral or risk-seeking or risk-avoiding. Assuming that the expected values of the sure option and the gamble are the same, the drift rate in System 2 depends on the DM’s risk attitude:

11 $$\begin{aligned} \mu _2 {\left\{ \begin{array}{ll}{=0} &{} \text {risk-neutral }\\ >0 &{} \text {risk-avoiding} \\ < 0 &{} \text {risk-seeking } . \end{array}\right. } \end{aligned}$$

#### Example 3

Loewenstein et al. (2015) used their dual-process approach (one-process, static, deterministic) to model inter-temporal choices, i.e., choices between rewards and punishments at different points in time.

As in the context of risky choices Loewenstein et al. (2015) assume that preferences in System 1 are constructed according to a motivational function *M*(*R*, *a*), which incorporates a variable *a* that captures the intensity of affective motivations (p. 59 Loewenstein et al., 2015), and is weighted by a function $$h(W,\sigma )$$ that reflects the willpower strength *W* and cognitive demands $$\sigma $$. For demonstration only two rewards are assumed, one immediate, $$R_I$$ and one delayed, $$ R_D$$, received at time $$T_D$$. The affective system motivational function (System 1) for this situation is defined as

12 $$\begin{aligned} M(R,a) = R_I +\exp ( -\delta _A(a) \cdot T_D)\cdot R_D \end{aligned}$$

where $\delta _A(a)$ is the discounting factor of an exponential discounting function for the affective system. Increased affective intensity *a* implies a smaller $\delta _A(a)$ (Loewenstein et al., 2015, p. 62). As before, *M*(*R*, *a*) is weighted by $h(W,\sigma )$. The exact form of this function is not specified in Loewenstein et al. (2015) but is meant to be decreasing in *W* and increasing in $\sigma$.

Preferences in System 2 are defined as

13 $$\begin{aligned} U(R)= R_I + \exp (-\delta _D\cdot T_D)\cdot R_D \end{aligned}$$

where $\delta _D$ is the discounting factor of an exponential discounting function for the deliberate system with $\delta _A(a) < \delta _D$.

As for the risky choice model Loewenstein et al. (2015) assumed that the functions (Eqs. 5, and 13) operate simultaneously, and the subjective value ${\mathcal V}(R)$ of an option is the sum of both processes:

14 $$\begin{aligned} {\mathcal V}(R) =U(R) + h(W, \sigma ) \cdot M(R,a). \end{aligned}$$

For implementing the approach to a dynamic dual process model Diederich & Zhao (2019) made the following specifications: Because *W* and $\sigma$ and the functional form of $h(W,\sigma )$ are not specified, $h(W,\sigma ):= h$, a constant, and $\delta _A(a):= \delta _1$ as the discounting factor for System 1. The mean difference in valence (drift rate) for the process in System 1 becomes

15 $$\begin{aligned} \mu _{1} = h\cdot [R_I-\exp ( -\delta _1 \cdot T_D) \cdot R_D]. \end{aligned}$$

Relabelling $\delta _D$ into $\delta _2$, operating in System 2 produces a mean difference in valence with

16 $$\begin{aligned} \mu _{2} = R_I-\exp ( -\delta _2 \cdot T_D) R_D. \end{aligned}$$

For details see (Diederich & Zhao, 2019).

Note that in the three examples operations in System 1 indirectly influence System 2, that is, the preference process in System 2 takes up where the preference process in System 1 terminates (i.e., the starting position for the second process). However, System 1 may influence System 2 also by allowing the equations defining drift rate $\mu _2$ to be a function of drift rate $\mu _1$ (for examples see Diederich & Trueblood, 2018).

## Dynamic-Stochastic Dual Process Framework: Architechtures

In the following, the dynamic-stochastic dual process model is shown for two different processing architectures. One version assumes serial procession with System 1 first, followed by System 2. The other version assumes a quasi parallel processing in which the two systems operate in parallel with crosstalk between them.

**Fig. 1:**
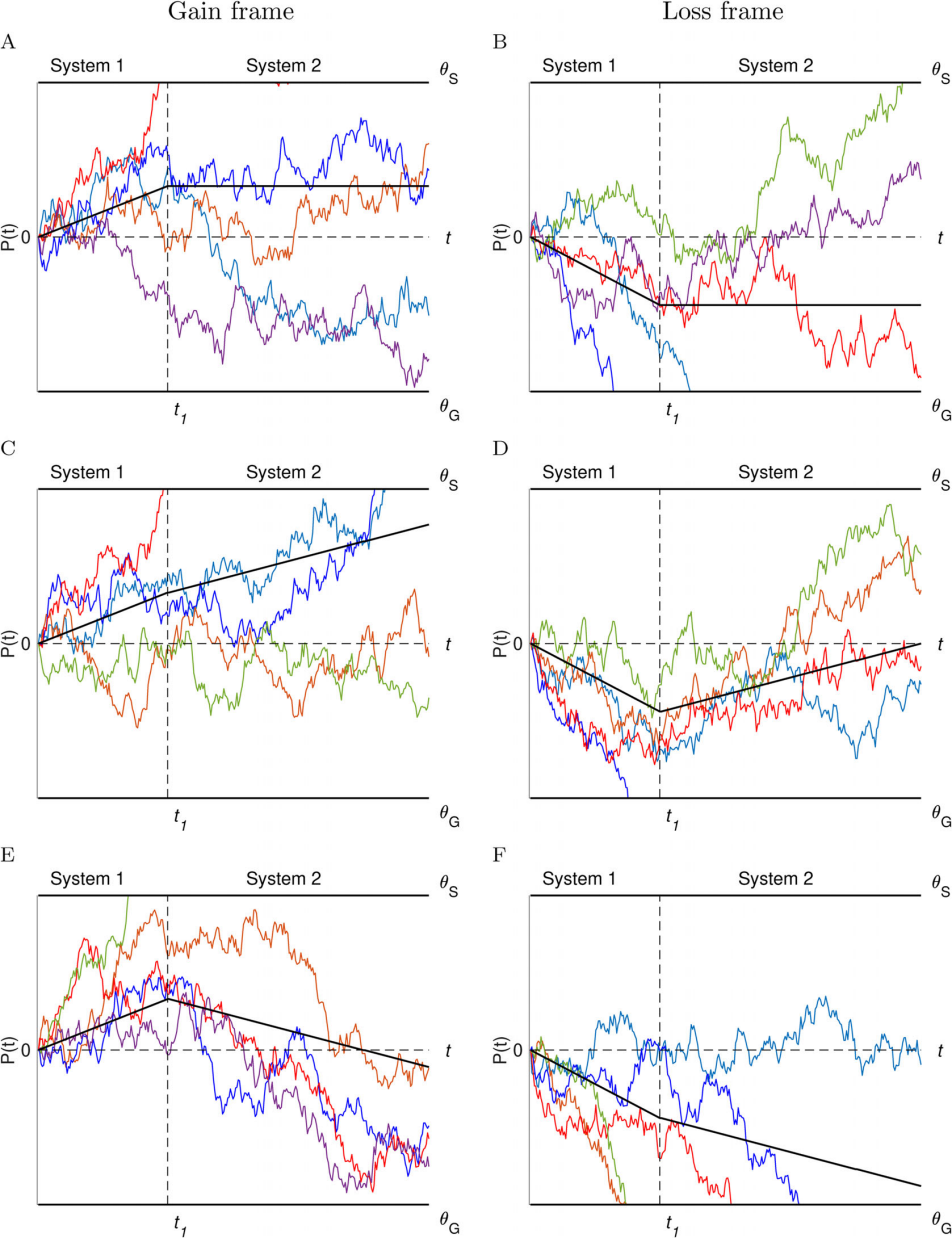
Two-stage dynamic-stochastic dual process model. Each trajectory symbolizes the preference accumulation for one trial (five trials in each panel). Once a trajectory hits a boundary, i.e., the criterion for choosing the sure option ($\theta _S$) or the gamble ($\theta _G$), a choice is made in favor of that option. The solid lines indicate the mean preference directed towards either criterion. The vertical dotted line marks the point in time when System 1 processing ends and System 2 processing starts. The left panels refer to a gain frame situation, and the right panels to a loss frame situation; the rows differ with respect to the risk attitude of the DM. **A**, **C**, **E** Gain frame—when operating in System 1, preference is, on average, in favor of choosing the sure option (mean valence $\mu _1 > 0$). **B**, **D**, **F** Loss frame—when operating in System 1, preference is, on average, in favor of choosing the gamble (mean valence $\mu _1< 0$). **A**, **B** Risk-neutral DM—when operating in System 2, the DM is indifferent between both choice options ($\mu _2 = 0$). **C**, **D** Risk-avoiding DM—when operating in System 2, the DM is in favor of choosing the sure option ($\mu _2 > 0$). **E**, **F** Risk-seeking DM—when operating in System 2, the DM is in favor of choosing the gamble ($\mu _2 < 0$)

### Two-Stage Dual Process Model

In the context of reasoning or preferential decision making, System 1 (intuitive, affective) is typically assumed to operate first and be responsible for producing various kinds of biases (see Kahneman, 2011, for an overview) before processing is taken over by System 2 (rational, deliberate) (e.g., Evans, 2008, for an overview). That means, at some point in time, the DM moves from operating mostly in System 1 to operating mostly in System 2. This is a serial or two-stage dual-process model.

These ideas are illustrated in Fig. 1 by referring to Example 2 in Section 2.3. This example is chosen because of its rich patterns of drift rates (Eqs. 10, and 11) and for ease of explanation. Each panel describes a different situation explained in the following. Within each figure a single-colored trajectory represents the preference accumulating (updating) process for one trial, i.e., when choosing between a sure option, *S*, and a gamble, *G*. The black solid lines represent the mean preference towards options *S* and *G*. The upper boundary $\theta _{S}$ refers to the criterion for choosing the sure option *S*, and the lower boundary $\theta _{G}$ to the criterion for choosing the gamble *G*. The left panels refer to the choice situations under a gain frame; the right panels to the ones under a loss frame. Consider the gain frame situation first. According to PT the DM is avoiding risk, and when operating in System 1, preference strength is building up towards $\theta _{S}$ for choosing the sure option (cf. Equation 10). At time $t_1$, operation switches from System 1 to System 2. In the examples, the drift rate in System 2 is determined by the risk attribute of the DM (Eq. 11). Once a trajectory hits a boundary the process stops and the response associated with the specific criterion is initiated. Note that the process can stop at any time while operating in System 1 or in System 2 to initiate a response. Consider, for instance, the first panels: for two trials a choice was initiated in favor of the sure options while operating in System 1 only. The preference processes in the loss frame (right panels) are analogous: according to PT the DM here is risk-seeking and while operating in System 1, preference strength is building up towards $\theta _{G}$ for choosing the gamble. When operating in System 2, the preference process is influenced by the risk attitude of the DM, the same as for the gain frame situation. Note that the switch from System 1 to System 2 was fixed here at time $t_1$ only for ease of communicating the ideas. Most likely the switching follows a distribution (Diederich & Oswald, 2014) as shown in the quantitative predictions in Section 3.1.2.

#### Qualitative Predictions

As mentioned in the introduction, dual process models account for a variety of cognitive biases. The two-stage dual process model makes specific predictions about the *size* of cognitive biases. Furthermore, the model makes predictions with respect to the choice probability/mean choice response time patterns. (The predictions assuming serial processing are indicated by **S**).

**S 1**: The size of the bias is a function of the time the DM operates in System 1.

**Fig. 2:**
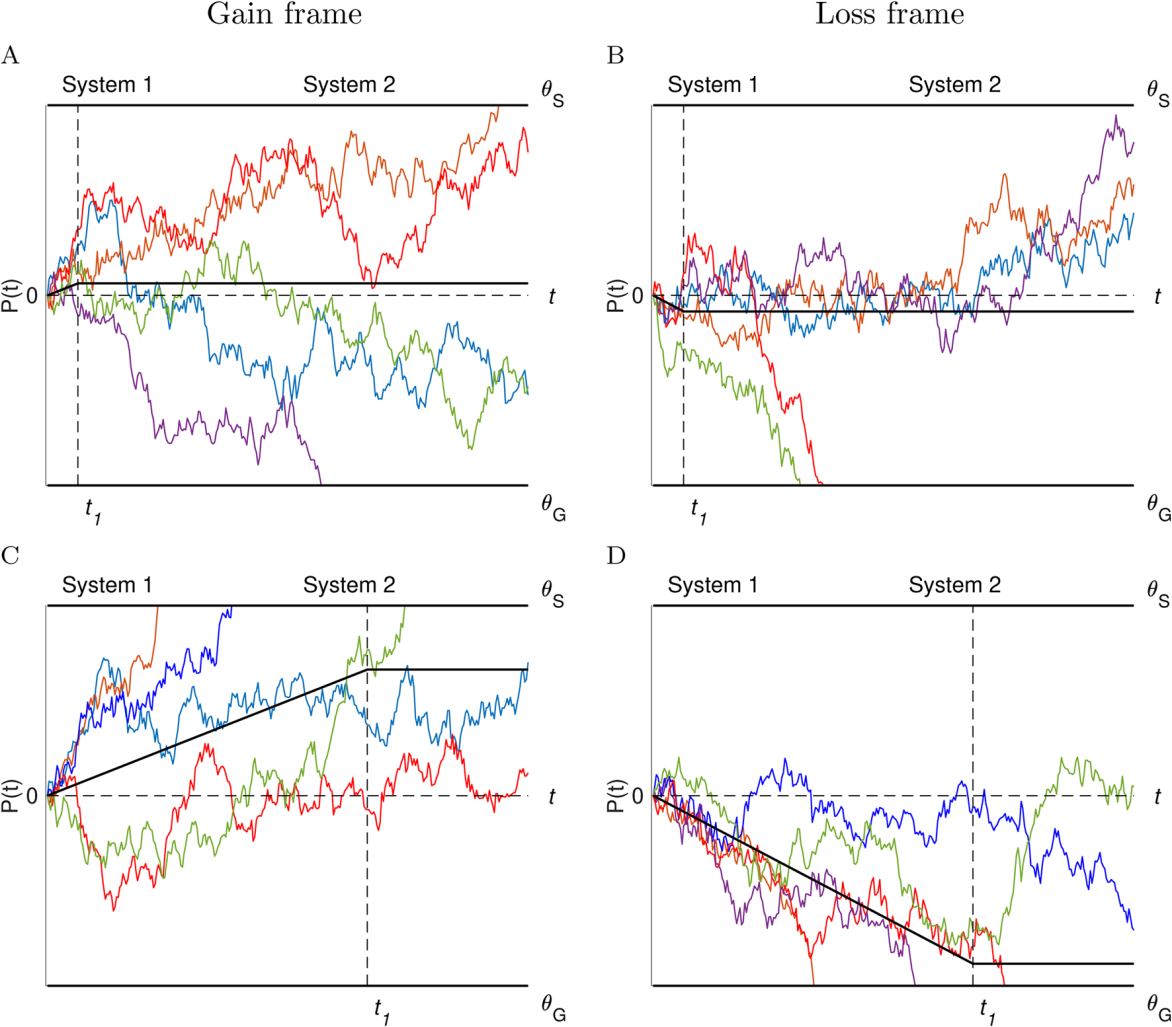
Two-stage dynamic-stochastic dual process model. For trajectory description see Fig. 1. **A**, **B** The time $t_1$ operating in System 1 is relatively short, and choices are mainly made when operating in System 2. **C**, **D** The time $t_1$ operating in System 1 is relatively long, and more choices are made when operating in System 1

Figure 2 illustrates the underlying ideas for the framing effect, defined as the difference in choice probability to choose the sure option or the gamble given a particular frame, i.e., $Pr(S|Gain) - Pr(G|Gain) \ne Pr(S|Loss) - Pr(G|Loss)$, or equivalently, $Pr(S|Gain) - Pr(S|Loss) \ne 0$. In each panel, each colored trajectory symbolizes the preference accumulation for one trial (five trials in each panel). The first row shows the two framing conditions assuming that a risk-neutral DM operates in System 1 only briefly before switching to System 2. In this case, the decision is mainly based on the rational process (System 2), and the framing effect should be small. That is, hardly any choices are made while operating in System 1, indicated by the number of trajectories hitting the criterion boundaries during that time. The longer the DM operates in System 1, the stronger the framing effect shown in the second row. Several choices have been executed during the first stage, and, depending on the frame, the sure option was chosen more often in the gain frame and the gamble was chosen more often in the loss frame. The same predictions are true for DM with risk-seeking and risk-avoiding attitudes.

**Fig. 3:**
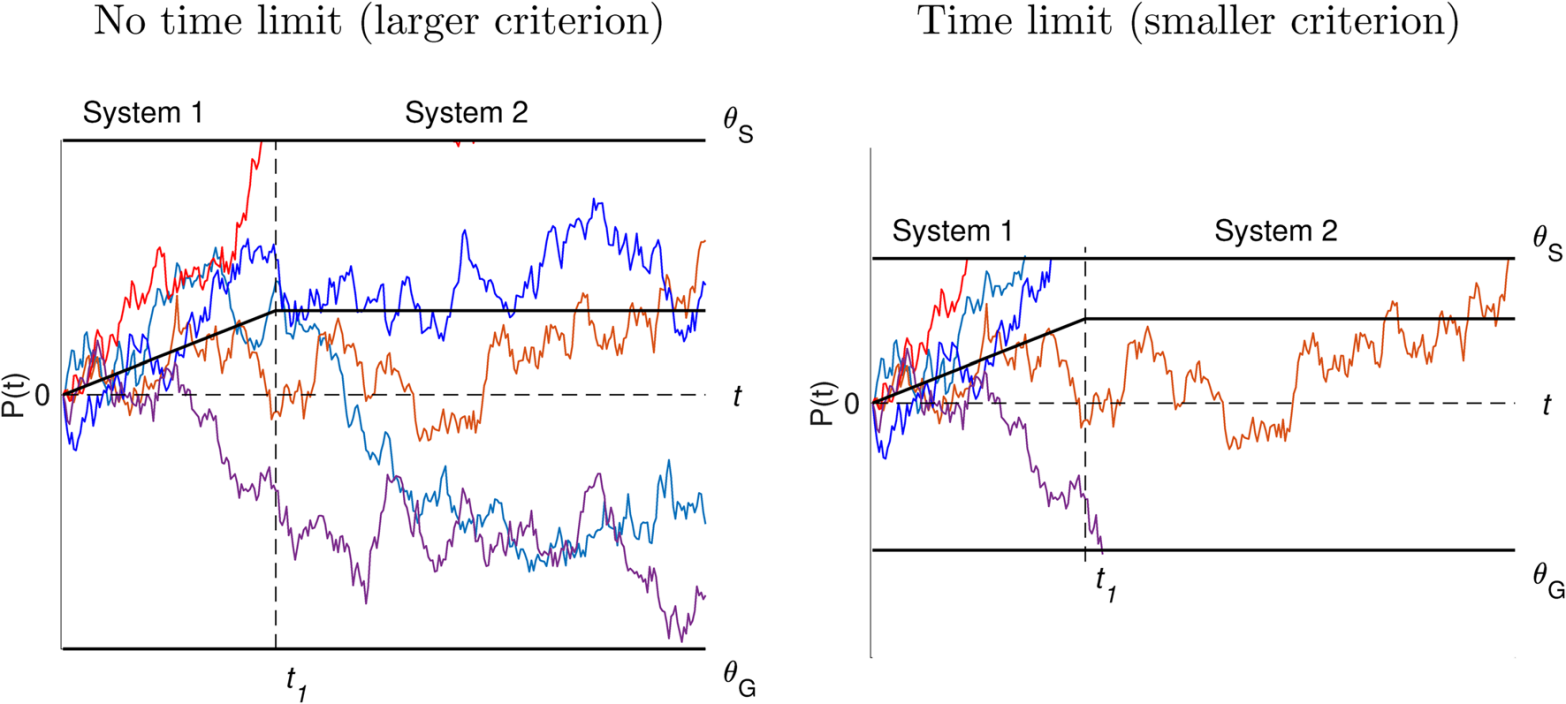
Two-stage dynamic-stochastic dual process model. For trajectory description see Fig. 1. Time constraints are reflected in the decision criteria. In the left panel, more time is available, increasing the boundaries; whereas in the right panel, less time is available, decreasing the boundaries

The decision criterion set by the DM is a function of time limits. With shorter time limits the criterion is set smaller. With a smaller criterion, more choice responses are initiated earlier. Because System 1 precedes System 2, more choices are made while operating in System 1.^4^

**S 2**: The size of the bias is a function of the time (limit) the DM has for making a choice.

Figure 3 shows the ideas for a risk-neutral DM under a gain frame; the same holds for a loss frame and DMs with other risk attitudes. As before, each of the five trajectories represents the preference accumulation process for one trial, i.e., to choose between the sure option and the gamble. The simulated trajectories are identical in both panels. The left panel shows the situation for larger criteria, for instance, no time pressure. The right panel shows the same situation but with time constraints. The set criteria are smaller, and a decision is reached sooner. In this example, three choices are made early on in favor of the sure option (the trajectories reached the criterion $\theta _S$).

Let us go back to Fig. 1 which describes a wide range of patterns about how preferences can be built in the two systems: in System 1 preferences are based on intuition and depend on the decision frame (gain/loss); in System 2 preferences are based on rationality/deliberation and depend also on the risk attitude of the DM. Even if this sounds very flexible, the two-stage dual process model makes predictions with respect to the choice probability/decision time patterns that provide a strong restriction to the model. These predictions, presented next, are generic to the two-stage model per se and are not specific to decision situations described here. If the mean valences (drift rates) in both systems do not point in different directions (as shown in Fig. 1A, B, C, and F) then the following patterns hold:

**S 3.1** If the mean valence in System 1 is larger (in absolute value) than in System 2, i.e., $|\mu _1|> |\mu _2|$, then the two-stage model **always** predicts faster mean response times to the more frequently chosen alternative.

This applies if the valences in System 1 are assumed to be stronger than the valences in System 2 for driving the process to the criteria to choose the sure option or the gamble. That is, the risk attitude triggered by the frames is stronger than the individual risk attitudes unrelated to any experimental manipulation. The numerical examples provided later assume this scenario.

**S 3.2** If the mean valence in System 1 is smaller (in absolute value) than in System 2, i.e., $|\mu _1|< |\mu _2|$, then the two-stage model **always** predicts faster mean response times to the less frequently chosen alternative.

This applies if the valences in System 1 are assumed to be weaker than the valences in System 2 for driving the process to the criteria to choose the sure option or the gamble. The psychological interpretation for **S 3.2** is that if a less favorable alternative is chosen in System 1, then the answer tends to be fast, before later on in System 2 the mean valence for the more favorable option even increases.

For a risk-seeking DM in a gain-frame condition (Fig. 1E) and a risk-avoiding DM in a loss-frame condition (Fig. 1D) the choice-probability/choice-response patterns are more complex but also consistent. If the mean valences (drift rates) in both systems point in opposite directions (as shown in Fig. 1A), then the following patterns hold:

**S 4.1** Preference reverses with increasing time spent operating in System 1. That is, the probabilities for choosing one alternative change from below (above) 0.5 to above (below) 0.5 as operating time in System 1 increases.

**S 4.2** The larger the mean valence in System 1 is as compared to System 2 (absolute values), the sooner the reversal occurs as a function of operating time in System 1.

**S 4.3** If the mean valence in System 1 is larger (absolute value) than in System 2, then the more frequently chosen alternative is chosen faster after the preference reversal.

**S 4.4** If the mean valence in System 1 is smaller than in System 2, then the less frequently chosen alternative is chosen faster after the preferences reversal.

That is, the same patterns as described before is observed after the probabilities for choosing the sure option and choosing the gamble crossed (0.5), and the reverse pattern is observed before the probabilities crossed. This will become clear when considering quantitative predictions.

#### Quantitative Predictions

The parameter values to show the quantitative predictions are as follows: $\mu _1 =.15$ for System 1 in a gain frame; $\mu _1= -.2$ for System 1 in a loss frame; $\mu _2= 0$ for a risk-neutral DM; $\mu _2=.1$ for a risk-avoiding DM; and $\mu _2=-.1$ for a risk-seeking DM. Note that the specific values are unimportant here. The relation between the parameters for System 1 follows the assumption of PT for which the value function for losses is steeper than for gains; $\mu _1 > \mu _2$ relates to prediction **S 3.1**. Predictions for **S 3.2** ($\mu _1< \mu _2$) are not considered here due to limited space. For simplicity, the time to switch from System 1 to System 2 follows a geometric distribution (Diederich & Oswald, 2014, for further distributions).

**Fig. 4:**
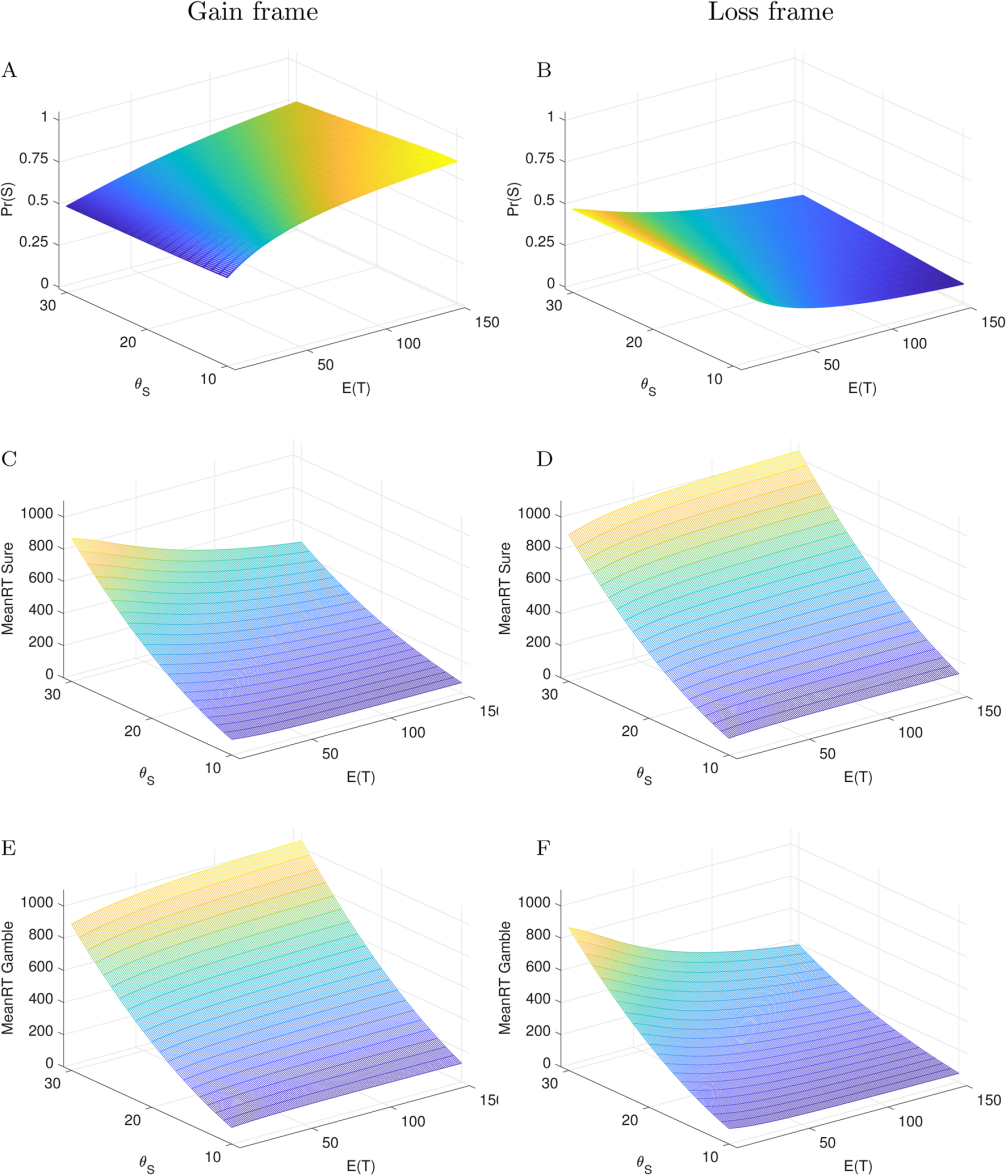
Two-stage dynamic-stochastic dual process model; Risk-neutral DM. Predicted choice probabilities for choosing the sure option in a gain frame (**A**) and a loss frame (**B**). Predicted mean choice response times for choosing the sure option in a gain frame (**C**) and a loss frame (**D**). Predicted mean choice response times for choosing the gamble in a gain frame (**E**) and a loss frame (**F**). On the *x*-axis is the expected time E(T) for operating in System 1 before switching to System 2. It follows a geometric distribution. On the *y*-axis is the criterion threshold $\theta _S$ for choosing the sure option

**Fig. 5:**
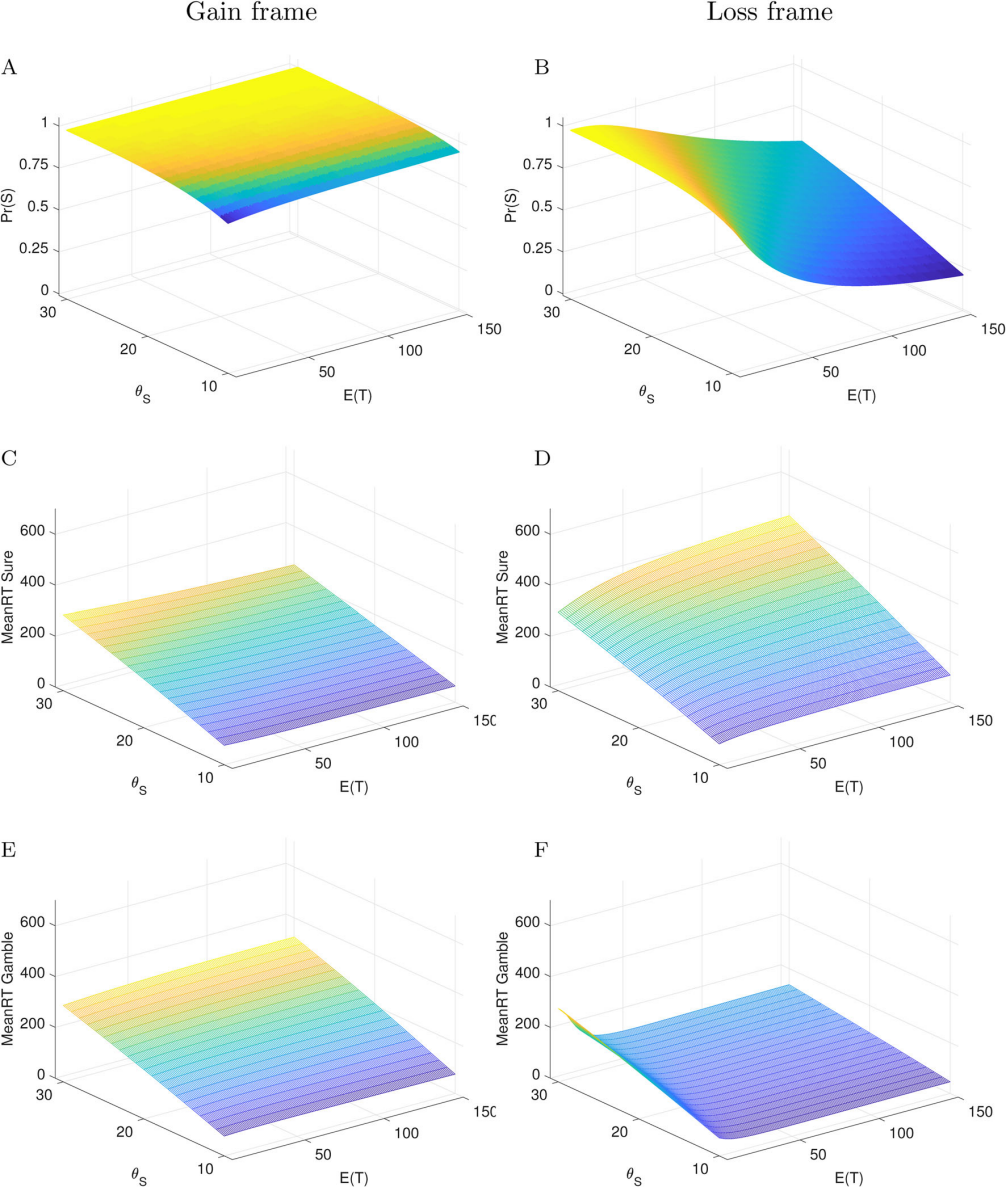
Two-stage dynamic-stochastic dual process model; Risk-avoiding DM. Predicted choice probabilities for choosing the sure option in a gain frame (**A**) and a loss frame (**B**). Predicted mean choice response times for choosing the sure option in a gain frame (**C**) and a loss frame (**D**). Predicted mean choice response times for choosing the gamble in a gain frame (**E**) and a loss frame (**F**). On the *x*-axis is the expected time E(T) for switching from System 1 to System 2. It follows a geometric distribution. On the *y*-axis is the criterion threshold $\theta _S$ for choosing the sure option

**Fig. 6:**
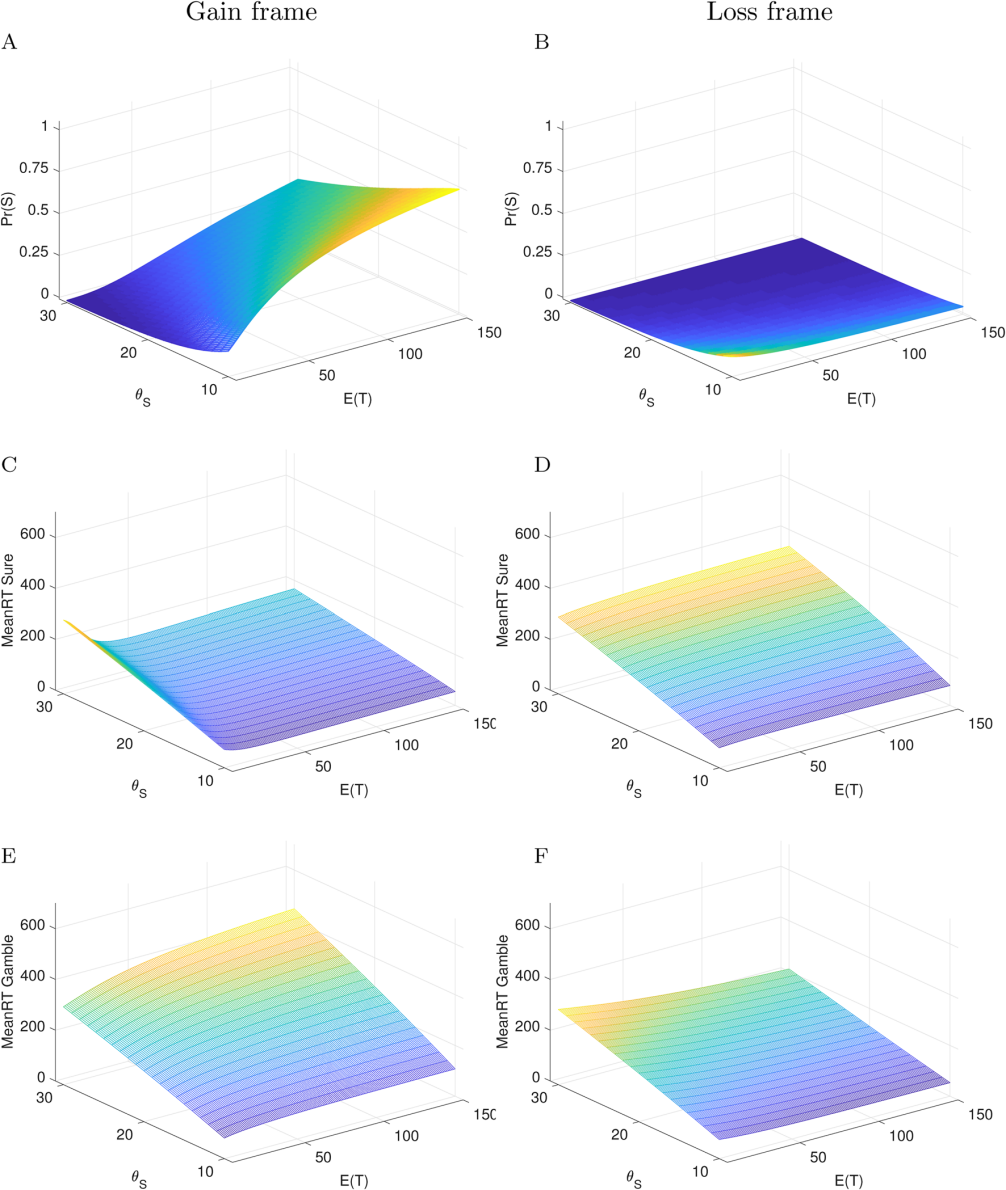
Two-stage dynamic-stochastic dual process model; Risk-seeking DM. Predicted choice probabilities for choosing the sure option in a gain frame (**A**) and a loss frame (**B**). Predicted mean choice response times for choosing the sure option in a gain frame (**C**) and a loss frame (**D**). Predicted mean choice response times for choosing the gamble in a gain frame (**E**) and a loss frame (**F**). On the *x*-axis is the expected time E(T) for operating in System 1 before switching to System 2. It follows a geometric distribution. On the *y*-axis is the criterion threshold $\theta _S$ for choosing the sure option

Figures 4, 5, and 6 show mesh plots of the predicted choice probabilities for choosing the sure option (top panels, A and B), the mean RT for choosing the sure option (middle panels, C and D), and the mean RT of choosing the gamble (bottom panels, E and F) as a function of the expected switching time, *E*(*T*), and the criterion $\theta$ ($\theta _S = -\theta _G$) for a gain frame (left panels) and a loss frame (right panels). Figure 4 shows it for a risk-neutral DM ($\mu =2=0$); Fig. 5 for a risk-avoiding DM ($\mu _2 >0$); and Fig. 6 for a risk-seeking DM ($\mu _2<0$).

Consider first prediction **S 1**: The size of the bias is a function of the time the DM operates in System 1 shown in panels A and B of each. The probability to choose the sure option in the gain frame *increases* as the time spent operating in System 1 increases before switching to System 2 (the expected time *E*(*T*)) (Panel A). On the other hand, the probability to choose the sure option in the loss frame *decreases* as the time spent operating in System 1 increases before switching to System 2 (Panel B), strengthening the overall framing effect, i.e. the bias. Note that the absolute size of the bias also depends on the DM’s risk attitude.

According to prediction **S 2**, the size of the bias is a function of the time (limit) the DM has for making a choice. Consider the risk-neutral DM first shown in Fig. 4A and B. Because the expected value of the sure option and the game are the same, a risk-neutral DM operating in System 2 is indifferent between both options, i.e., the probability to choose the sure option or the gamble is 0.5. With increasing criteria $\theta$ the choice probabilities approach this value. It depends on *E*(*T*) how fast and close this occurs. The framing effect is defined as the difference in choice probability to choose the sure option or the gamble given a particular frame. When comparing the probabilities in both panels across the respective *E*(*T*) and $\theta$ it becomes obvious that the difference *increases* with increasing *E*(*T*) and increasing $\theta$, and thus the bias (framing effect) becomes *larger*.

The risk-avoiding DM (Fig. 5A and B) prefers risk-less options. In the gain frame, the probabilities approach 1 for choosing the sure option with an increasing criterion because the valences in both systems point in the same direction (cf. speed-accuracy). In the loss frame, the probabilities for choosing the sure option also increase but are set off by System 1. Again, when comparing the probabilities in both panels across the respective *E*(*T*) and $\theta$ it becomes obvious that the difference between choice probabilities *decreases* with increasing *E*(*T*) and increasing $\theta$, and thus the bias (framing effect) becomes *smaller*. The same reasoning holds for the risk-seeking DM for choosing the gamble (Fig. 6). A comparison of Panel A and B shows that the difference between choice probabilities *decreases* with increasing *E*(*T*) and increasing $\theta$, and thus the bias (framing effect) becomes *smaller* here as well.

The two-stage dynamic stochastic dual model predicts a coherent choice-probability/choice-response time pattern and thereby provides a strong test of the model. Prediction 3.1 is concerned with the pattern when mean valences in both systems do not point in different directions for choosing the sure option or the gamble. This prediction is shown in Fig. 4 for both frames, in Fig. 5 for the gain frame, and in Fig. 6 for the loss frame. Take as an example the risk-neutral DM ($\mu _2 = 0$). In the gain frame, the probabilities for choosing the sure option are larger than 0.5, and the mean choice response times for choosing it are smaller than for choosing the gamble. In the loss frame, the probabilities for choosing the sure option are smaller than 0.5, and the mean choice response times for choosing the sure option are longer than for choosing the gamble. Prediction 3.2 is not shown here but see (Diederich & Oswald, 2014; Diederich, 2016). Predictions S 4.1 to S 4.4 are concerned with the patterns when mean valences in System 1 and System 2 point in opposite directions for choosing between options. This is the case for a risk-avoiding DM in a loss frame (Fig. 5B) and a risk-seeking DM in a gain frame (Fig. 6A). That is, in both cases, preference reversals occur as a function of *E*(*T*). For the risk-avoiding DM the model predicts fast responses for the less frequently chosen alternative *after* a preference reversal occurs and fast response for the more frequency chosen alternative *before* the preference reversal occurs (Fig. 5B, D, F). For the risk-seeking DM it is the opposite pattern: fast responses for the less frequently chosen alternative *before* a preference reversal occurs and fast responses for the more frequently chosen alternative *after* the preference reversal occurs (Fig. 6A, C, E).

A brief comment on two-stage (dual) process model for perceptual decision making. The notion of dual processes here have sometimes a slightly different meaning, also called two-stage models and are closely related to specific experimental paradigm in which stimulus information is provided sequentially with different onset times. For instance, the time-window of integration (TWIN) model assumes a race between two (or more) processes from different modalities (e.g. auditory, visual, tactile) in a first stage and depending which of the processes wins and opens a so-called window of integration may trigger a facilitated response or not in the second stage (e.g., Diederich & Colonius, 2008, 2015, 2019). Specifically, the processes in the first stage are modeled by assuming exponential distributions, the second stages assumes a normal distribution. The model parameters are related to stimulus intensities, temporal and spatial arrangements.

Salinas and colleagues have developed a paradigm for separating the process of perceptual decision making from motor planning and execution (Stanford et al., 2010; Salinas et al., 2010, 2014; Shankar et al., 2011). Their accelerated race-to-threshold model assumes two stages. In a first stage, two variables race against each other to a threshold, at which a saccade is initiated. Each variable represents the motor plan to perform a saccade to one of the two spots, and the variable that first crosses the threshold determines the spot to which the saccade goes. In a second stage, the incoming perceptual information differentially modulates the trajectories toward it: the variable corresponding to the correct target accelerates and the one corresponding to the incorrect distractor decelerates. The underlying processes assume (truncated) bivariate Gaussian distributions. The model includes at least 10 free parameters.

**Fig. 7:**
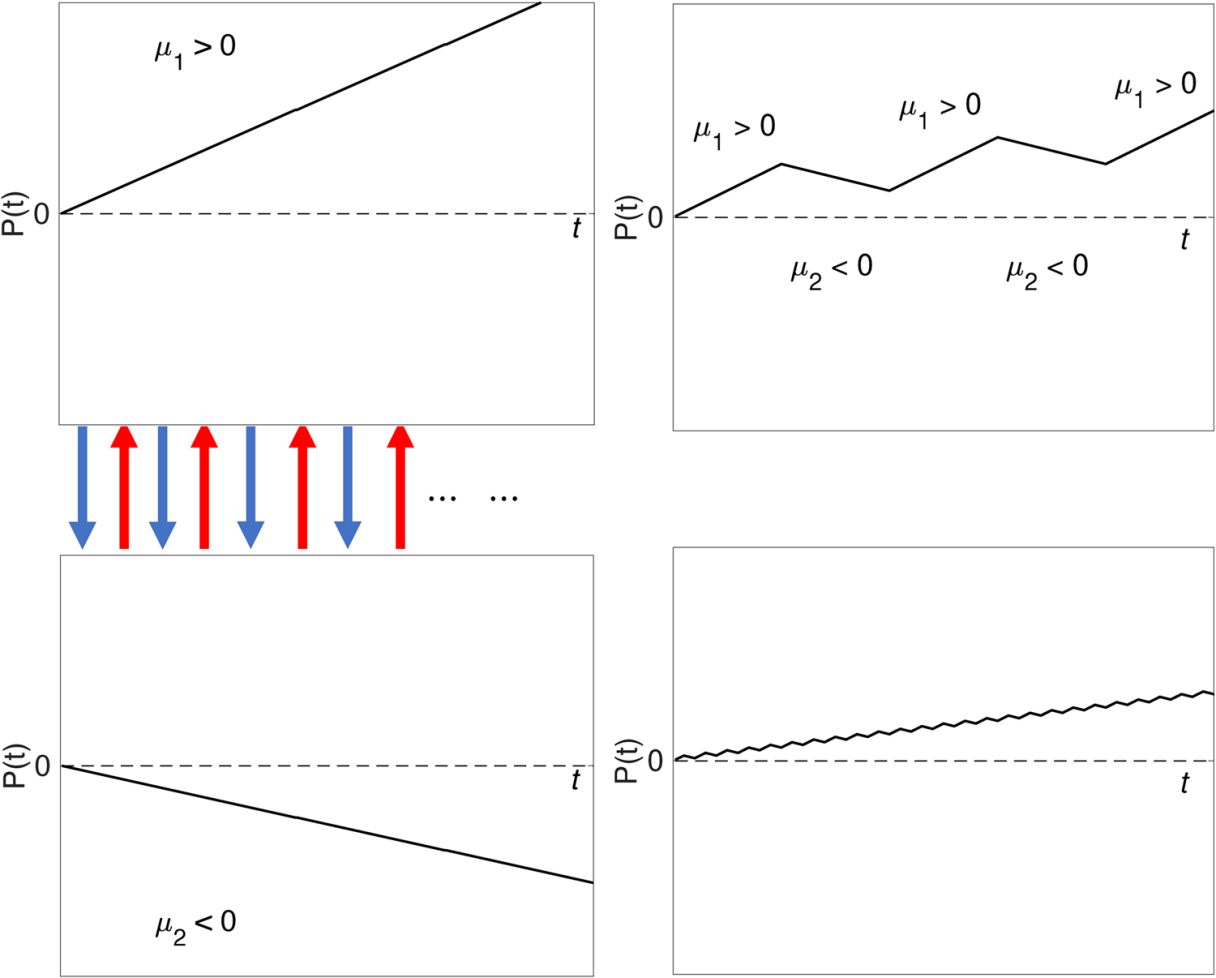
Parallel dynamic-stochastic dual process model. For details see text

**Fig. 8:**
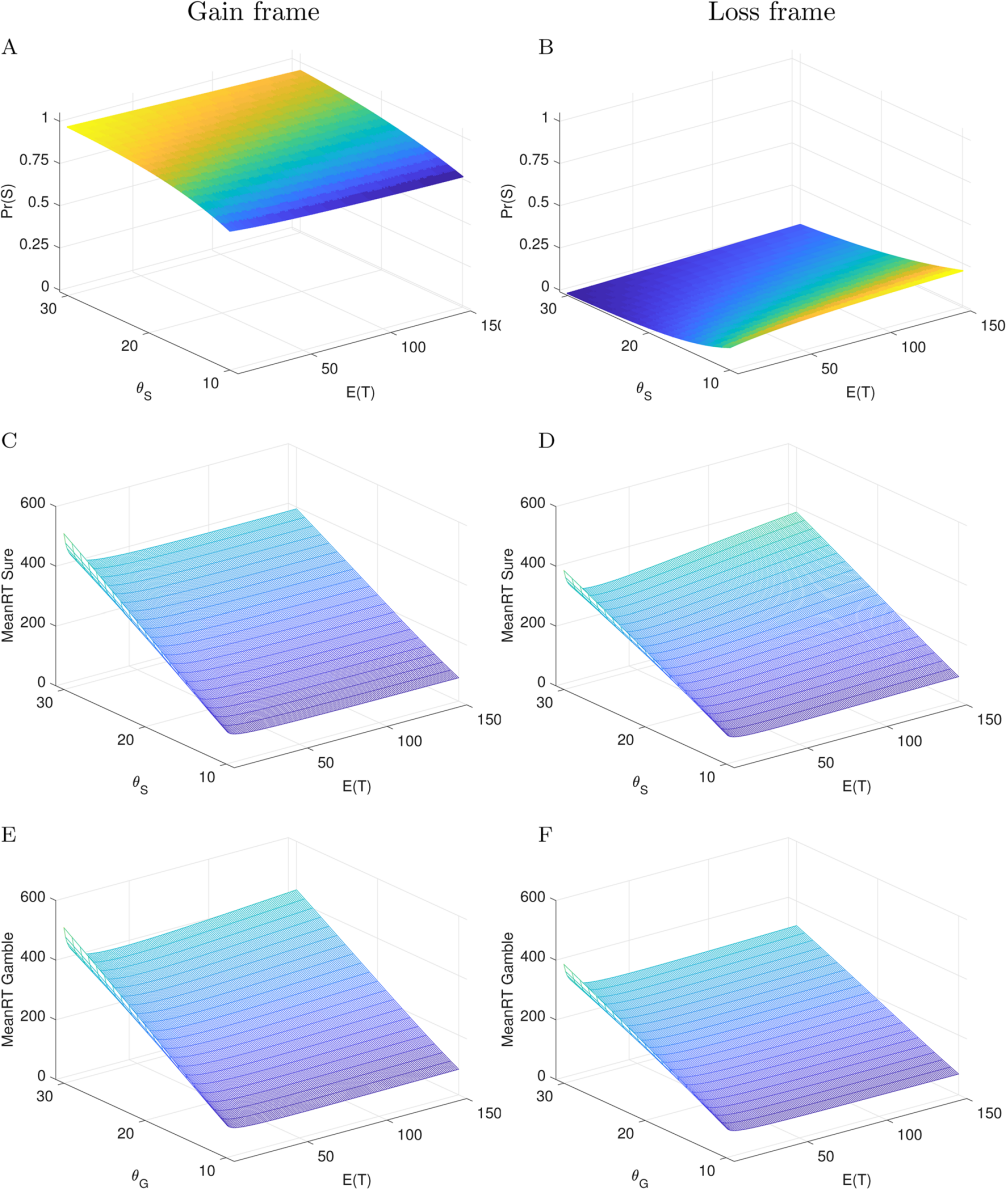
Parallel dynamic-stochastic dual process model starting with any system (probability 0.5); risk-neutral DM. Predicted choice probabilities for choosing the sure option in a gain frame (**A**) and a loss frame (**B**). Predicted mean choice response times for choosing the sure option in a gain frame (**C**) and a loss frame (**D**). Predicted mean choice response times for choosing the gamble in a gain frame (**E**) and a loss frame (**F**). On the *x*-axis is the expected time E(T) for operating in System 1 before switching to System 2 and the expected time for operating in System 2 before switching back to operating in System 1. It follows a geometric distribution. For simplicity, the expected times are assumed to be identical. On the *y*-axis is the criterion threshold $\theta _S$ for choosing the sure option

**Fig. 9:**
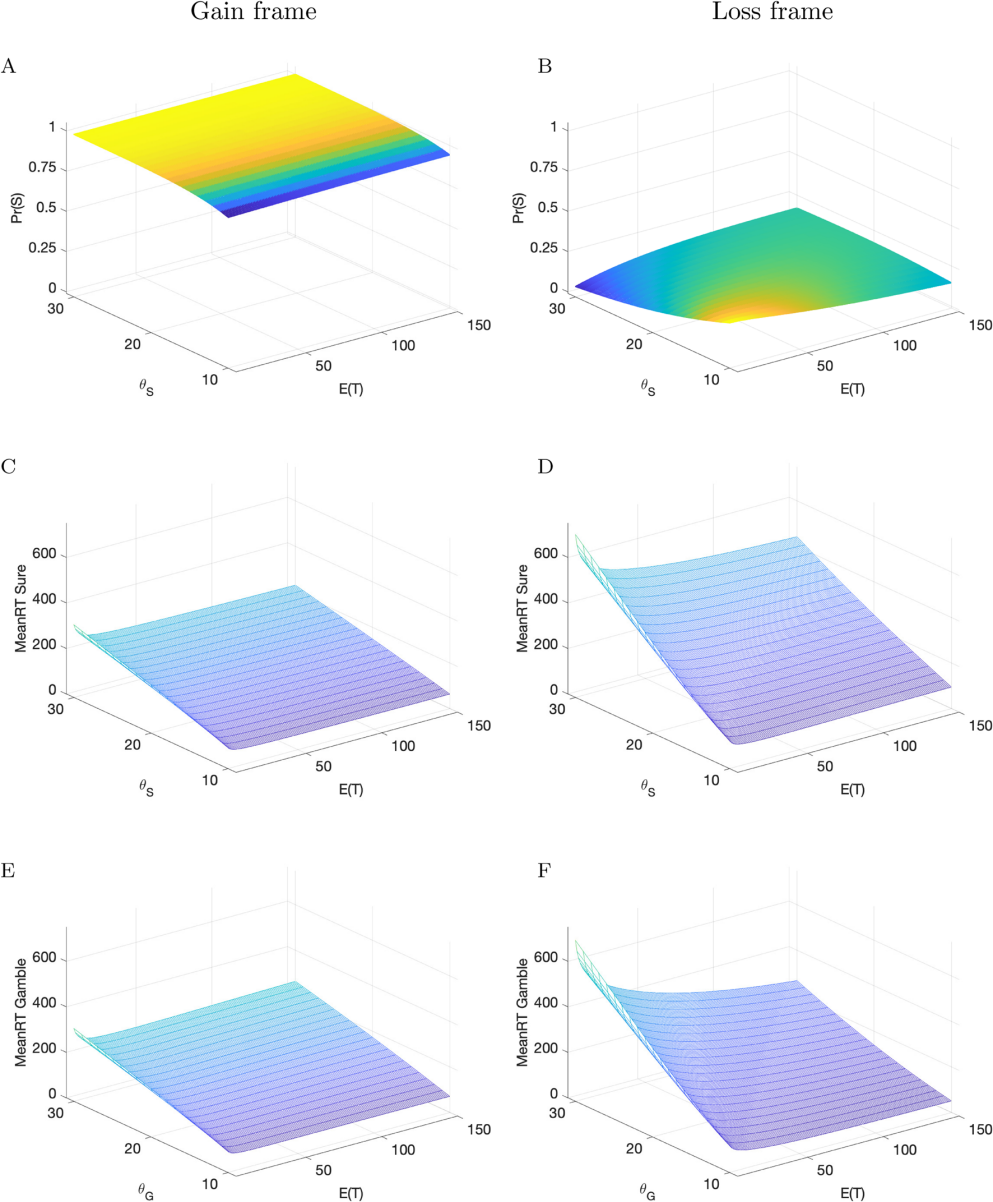
Parallel dynamic-stochastic dual process model starting with any system (probability 0.5); risk-avoiding DM. Predicted choice probabilities for choosing the sure option in a gain frame (**A**) and a loss frame (**B**). Predicted mean choice response times for choosing the sure option in a gain frame (**C**) and a loss frame (**D**). Predicted mean choice response times for choosing the gamble in a gain frame (**E**) and a loss frame (**F**). On the *x*-axis is the expected time E(T) for operating in System 1 before switching to System 2 and the expected time for operating in System 2 before switching back to operating in System 1. It follows a geometric distribution. For simplicity, the expected times are assumed to be identical. On the *y*-axis is the criterion threshold $\theta _S$ for choosing the sure option

In the context of visual perception with targets and distractors, Devaine et al. (2014) assumed a race between intentional and stimulus-driven processes in the first stage, and an interaction between both process in the second stage, similar to the accelerated race-to-threshold model. They assume lognormal distributions. The model includes at least 7 free parameters.

Diederich & Colonius (2021) showed that the two-stage dynamic model can account for the data at least as well as the accelerated race-to-threshold model by including only three free parameters. Note that the experiments were done with monkeys providing thousands of trials and therefore, the models could even be fitted to the entire distributions.

### Parallel Dynamic-Stochastic Dual Process Model

The sequential processing assumption is very influential in dual process theories of decision-making. According to Kahneman & Frederick (2002) the fast System 1 (driven by heuristics) yields a default intuitive response that can be modified by the rational System 2. This “default-interventionist’’ assumption (Evans, 2008, p.266) implies a sequential ordering of the two systems with System 1 starting first and System 2 intervening later. In contrast, Sloman (1996) suggested that the systems operate in parallel and are competitive. A “parallel-competitive” (Evans, 2008, p.266) approach can be modeled as a race model, i.e. a race takes place between the processes in System 1 and System 2, and the winner of the race determines the response. Given, however, that System 1 is much faster than System 2 (e.g., Kahneman, 2011), System 1 would win the race in almost all cases.^5^ Then, as Diederich and Trueblood (2018) pointed out, all responses are determined by processes operating in the intuitive System 1, making System 2 irrelevant (see also Alós-Ferrer, 2018, for a critical evaluation of race models in this context).

An alternative approach is to assume a stochastically dependent race between the systems. This requires a two-dimensional Wiener process (rather than the one-dimensional utilized throughout here). N-dimensional Wiener processes have been proposed recently for multi-alternative choice situations (Mallahi-Karai & Diederich, 2019, 2021), and dependencies between the two processes may be modeled via covariances between the different processes. This requires determining the specific dependencies (and justification of positive or negative correlations between the racers) and is not pursued here.

Yet another alternative approach is to assume a fast switching back and forth between the two systems and thereby producing pseudo-parallel processing with dependency. This is pursued in the remainder here; Fig. 7 shows the basic ideas. The left-hand side of the figure shows the two processes, both starting at the same time. The arrows between the two displays indicate switching back and forth. The right-hand side shows the effect of two different switching frequencies between the two processes, lower in the upper display and higher in the bottom display.

Assuming the same operation time distributions in both systems the switching frequency most obviously depends on the expected value of the distributions, that is, the expected time (the mean of the distribution) for operation in each system. Small expected values produce high switching frequencies whereas large expected values produce low switching frequencies. The underlying distributions and their expected values, called *time schedule* (Diederich & Oswald, 2014) (see digression below) do not have to be the same for both systems. Indeed, the importance or impact of one system in the decision process can be related to the assumed distributions and/or to their expected values. However, one can argue that a model closest to a parallel model should have the same distributions and expected values in both systems, as illustrated in Fig. 7.

The two-stage dual process assumes that operating in System 1 occurs before switching to operating in System 2. For the parallel architecture it is possible that either System 1 comes first (as shown as an example in Fig. 7), or System 2 comes first, or both systems have the same (or specified) probability for operating first.^6^ Again, one can argue that a model closest to a parallel model should have the same probability for operating first. The order in which the systems are considered is called *order schedule* (Diederich & Oswald, 2014) (see digression below). Note that both, time and order schedules are parameters of the model and are briefly discussed in the digression.

Next, the overall direction of the preference process is determined by the system with the larger drift rate. Recall that in the framing example here, the absolute value of the drift rate in System 1 was assumed to be larger than in System 2. That is, in the gain frame, the tendency to choose the sure option is always larger than to choose the gamble, regardless of the risk attitude of the DM. Similarly, in the loss frame, the tendency to choose the sure option is always smaller than to choose the gamble, regardless of the risk attitude of the DM. This should be independent of the assumed order schedule.

When assuming the same operational time distributions with identical expected values and fixing the probability of the order in which the two systems operate, the serial and the parallel versions of the model have the same number of parameters.^7^ However, some qualitative predictions for the parallel model seem to be more difficult to derive, and we need to rely more on quantitative predictions shown next. Figures 8 and 9 show the quantitative predictions separately for the three risk attitudes assuming that any system comes in first. The predictions assuming the process starts operating in System 1 or assuming the process starts with System 2 are both provided in the supplement.

The operating times in each system (switching frequency) have less influence on the patterns of choice probabilities as compared to the ones in the serial model version. The larger drift rate (here the one in System 1) determines the direction of the preference process towards the choice alternatives. In the gain frame, the sure option is the more preferred alternative and in the loss frame the gamble is the more preferred option; the risk attitude plays no role in this pattern. No preference reversals occur as a function of operating times in both systems (switching frequencies). Based on this the following hypotheses are proposed:

**P 1.** The time the DM operates in System 1 and System 2 has no/little effect on the size of the bias.

Recall that the size of the framing effect is defined as the difference in choice probability to choose the sure option or the gamble given a particular frame. No effect implies that $Pr(S | Gain)-Pr(S | Loss) = constant.$ Although the choice probabilities do not change substantially as a function of *E*(*T*) as compared to the serial architecture, it is difficult to judge directly from the figures whether the size of the bias changes as a function of *E*(*T*) or not. Figure 11 shows a plot of the difference between the probability to choose the sure option in a gain frame and in a loss frame for two criterion bounds ($\theta =10$ left panels; $\theta = 30$ right panels) as a function of *E*(*T*). The black dashed lines refer to the result assuming parallel processing of the two systems revealing indeed little or no changes in bias size. For comparison, the red lines show the size of the bias assuming the systems operate in a sequence. Obviously, in the latter case, the size of the bias is strongly affected by operating times.

**Fig. 10:**
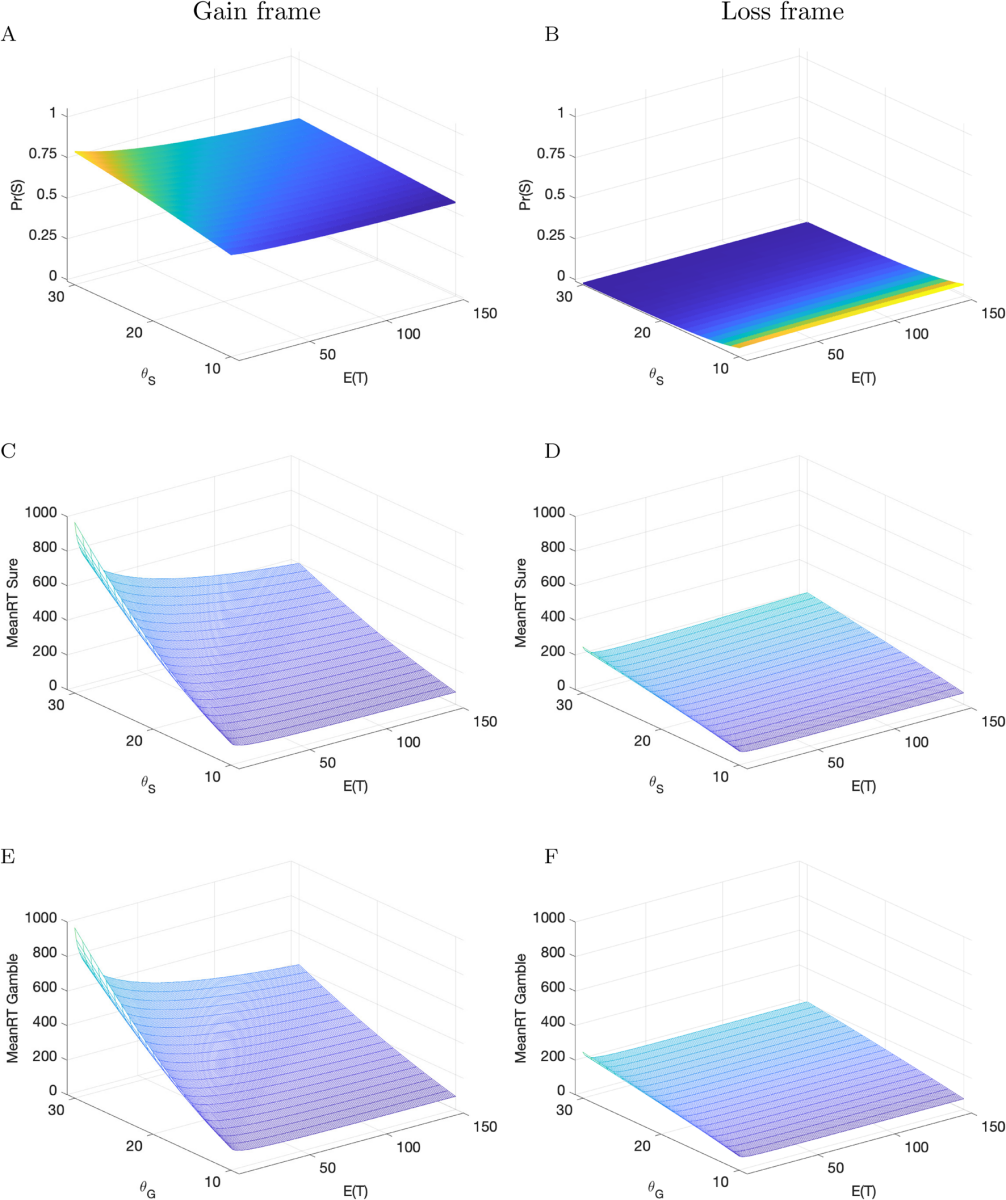
Parallel dynamic-stochastic dual process model starting with any system (probability 0.5); risk-seeking DM. Predicted choice probabilities for choosing the sure option in a gain frame (**A**) and a loss frame (**B**). Predicted mean choice response times for choosing the sure option in a gain frame (**C**) and a loss frame (**D**). Predicted mean choice response times for choosing the gamble in a gain frame (**E**) and a loss frame (**F**). On the *x*-axis is the expected time E(T) for operating in System 1 before switching to System 2 and the expected time for operating in System 2 before switching back to operating in System 1. It follows a geometric distribution. For simplicity, the expected times are assumed to be identical. On the *y*-axis is the criterion threshold $\theta _S$ for choosing the sure option

Because the choice probabilities increase/decrease as a function of the decision criterion, the framing effect becomes larger with increasing decision thresholds. This is confirmed by the quantitative predictions shown in Figs. 8, 9, and 10 by comparing the A and B panels.

The last prediction is concerned with the choice probability/time pattern. It is well known that a Wiener process with drift without additional assumptions (e.g., a priori bias, see Ratcliff et al. (2016) for a recent overview) predicts the same mean response times for both choice options. However, the current quasi-parallel dynamic-stochastic dual process model is a non-time homogeneous process (Diederich, 1997; Diederich & Oswald, 2014) and therefore, the mean response times should differ.

**P 3.** If the absolute value of the drift rate in System 1 is larger than in System 2, then the mean response time for the more frequently chosen alternative should be (slightly) smaller than the mean response time for the less frequently chosen alternative, regardless of *E*(*T*), $\theta$, and risk attitude.

As can be seen in Figs. 8, 9, and 10 the mean response times are very similar for choosing the sure option and the gamble within a given frame (compare panel C to panel E for the gain frame and panel D to panel F for the loss frame) but not identical.

## Testing the Models

The models are tested on a set of data from (Guo et al., 2017). Participants (n = 195) made decisions between gambles and sure options. The sure options were either framed as gains or losses. The experiment consisted of two blocks; they differed in the amount of time participants were given to make a choice. One block was a time pressure (TP) block where participants were given 1000 ms to make a choice. The other block was a no time pressure (noTP) block where participants could take as much time as they wanted to respond. Each block consisted of 72 gain frame and 72 loss frame trials. At the start of each trial, participants were given an initial amount of money selected randomly from a uniform distribution ranging from $20 to $90. Similar to De Martino et al. (2006), participants chose between a sure option to keep a portion of the initial amount and a gamble to possibly keep the entire initial amount. The probabilities of winning the gamble were created by randomly drawing 72 probabilities from a pool of three normal distributions (m = 0.28, 0.42, and 0.56; std = 0.2). The initial amounts and probabilities of winning the gamble were randomly paired together to form 72 unique test trials. From these pairs, the sure option for each trial was created to match the expected value of the gamble, depending on frame. The options were presented as color-coded pie charts.

For the model fitting, we grouped responses into three levels of initial amount (with means of $32, $56, and $79) and three levels of probabilities of winning (with means of 0.28, 0.42, and 0.56). This produced nine different stimulus categories. For each of the nine categories, there were gain/loss conditions and time pressure / no time pressure conditions. In total, we had 9 $$\times $$ 2 (frame) $$\times $$ 2 (time pressure) = 36 choice probabilities. For each of the 36 conditions, we also had mean response times for selecting the gamble and mean response times for selecting the sure option. Thus, in total, we used 108 data points for model fitting.

The procedure for estimating model parameters follows one example from Diederich and Trueblood (2018), assuming that preferences in System 1 are constructed according to prospect theory (Kahneman & Tversky, 1979) and preferences in System 2 according to expected utility theory (vonNeumann & Morgenstern, 1953).

Consider System 1 first. The value $\mathcal {V}$ of a simple prospect that pays *x* with probability *p* (and nothing otherwise) is given by:

17 $$\begin{aligned} \mathcal {V}(x,p)= w(p) v(x), \end{aligned}$$

where *w* measures the impact of probability *p* on the attractiveness of the prospect and *v* the subjective value of the consequence *x*. With

18 $$\begin{aligned} w(p) = \frac{p^\gamma }{(p^\gamma +(1-p)^\gamma )^{1/\gamma }} \end{aligned}$$

the probability weighting function *w*(*p*) produces an inverse S-shaped function for $\gamma < 1$, accounting for overweighting low probabilities and underweighting moderate to high probabilities. In the current example, we have four different given probabilities: 0.28, 0.42, 0.56, and 1.00 (for the sure option). With

19 $$\begin{aligned} v(x) = {\left\{ \begin{array}{ll}x^{\alpha } &{} \text {if } x \ge 0 \\ -\lambda |x|^{\beta } &{} \text {if } x < 0 \end{array}\right. } \end{aligned}$$

the value function *v*(*x*) is concave for gains, convex for losses, and steeper for losses than for gains, accounting for risk-averse behavior in the gain domain and risk-seeking behavior in the loss domain. For the current example, we have three given values: 32, 56, and 79.

To implement the static-deterministic PT into the dynamic-stochastic framework assume that, under a gain frame, preference evolves in System 1 by comparing the consequences when choosing the gamble and the sure option, producing valence values ${V}^G(t)$ and $V^{S_{gain}}(t)$ at any moment in time. The expected values of the valences are related to the PT values such that $E[{V}^G(t)] = \mathcal {V}_G\cdot t$ and $E[{V^{S_{gain}}}(t)] = \mathcal {V}_{S_{gain}}\cdot t$, resulting in a difference in mean valence (the drift rate)

20 $$\begin{aligned} \mu _{1_{gain}} = \mathcal {V}_G -\mathcal {V}_{S_{gain}}. \end{aligned}$$

An analog reasoning applies to decision making under a loss frame, resulting in a difference in mean valence

21 $$\begin{aligned} \mu _{1_{loss}} = \mathcal {V}_G -\mathcal {V}_{S_{loss}}. \end{aligned}$$

The given probabilities and amounts to win/lose are inserted in Eq. (18) and (19), the parameters $\alpha , \beta , \gamma$, and $\lambda$, estimated from the data form the mean valences and eventually determine the drift rates for the System 1. Note that the underlying decision models drives the process, and the drift rate is estimated indirectly from the data by estimating the parameters of the respective decision model.

**Fig. 11:**
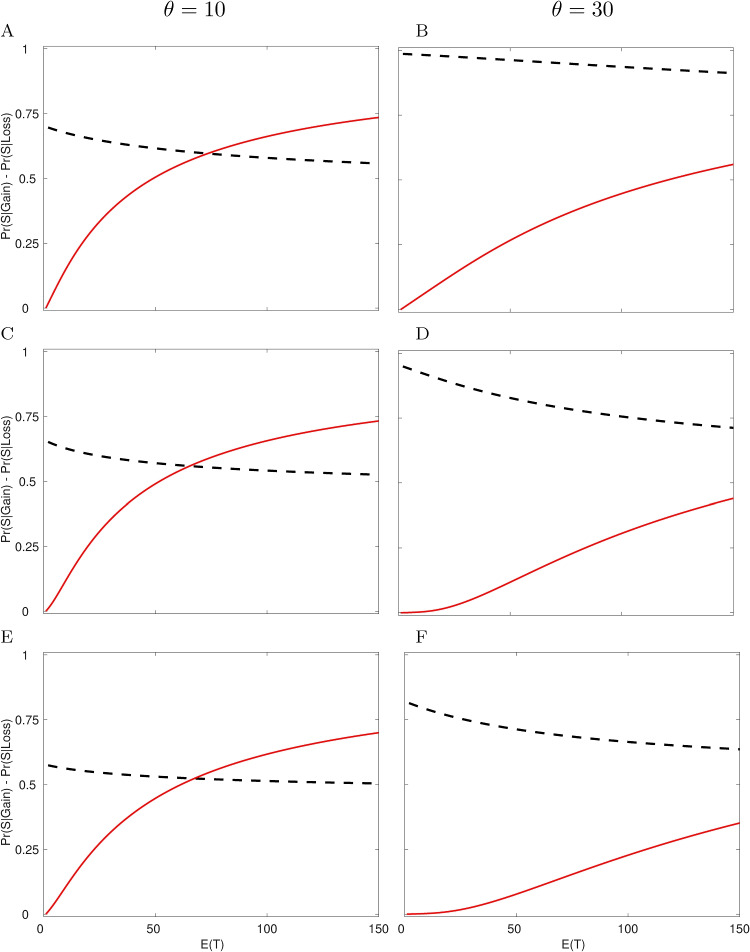
Size of the biases defined by the difference in probability to choose the sure option in different frames. The columns refer to different decision criteria; the rows to the three risk attitudes (neutral; risk-avoiding; risk-seeking; from top to bottom). The black dashed line refers to a parallel architecture assumption; the red line to a sequential architecture. Note that E(T) on the *x*-axis refers to either the expected time operation in System 1 before switching to System 2 (sequential) or to the expected time operating in either system (parallel)

**Fig. 12:**
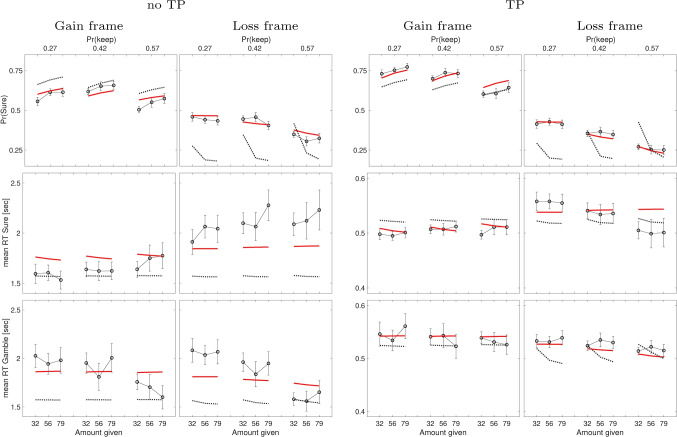
Model accounts for the Guo et al., 2017 data. The left column refers to the no-time-limit condition, the right column to the time pressure condition. The first row shows the gain frame condition, the second row the loss frame condition. The black lines show the data including confidence intervals. The red lines represent the account of the two-stage dual process model (serial architecture). The black dotted lines represent the account of the quasi-parallel dual process model assuming that the process starts in any of the two systems with equal probability and having the same operating times in both systems

**Fig. 13:**
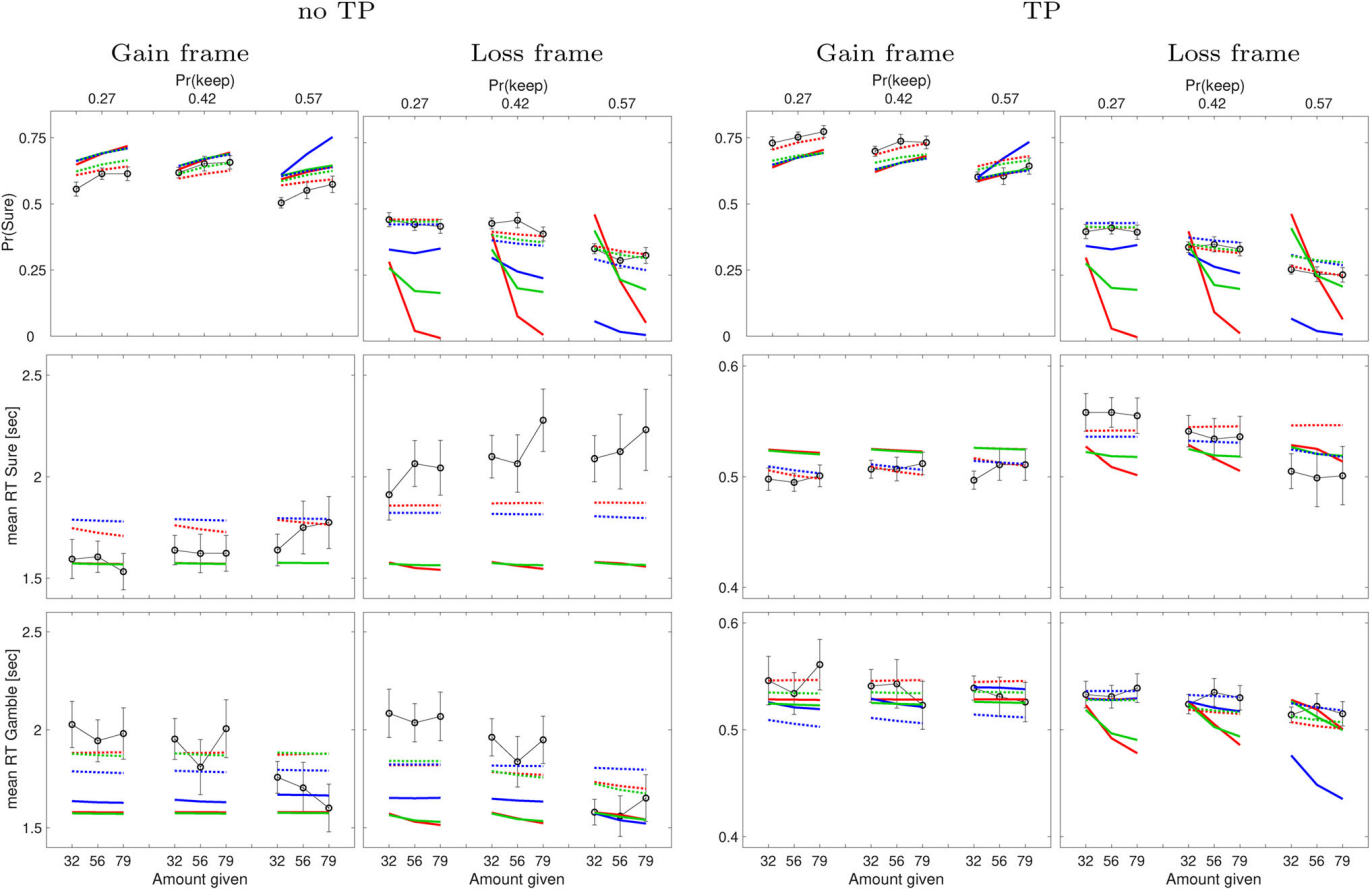
Model accounts for the Guo et al., 2017 data. The left column refers to the no-time-limit condition, the right column to the time pressure condition. The first row shows the gain frame condition, the second row the loss frame condition.The black lines show the data including confidence intervals. All model versions assume quasi-parallel processing of the two systems In particular, $1/r_{1_{gain}} = 1/r_{2_{gain}}$ and $1/r_{1_{loss}} =1/r_{2_{loss}}$ (solid lines) or $1/r_{1_{gain}} \ne 1/r_{2_{gain}}$ and $1/r_{1_{loss}} \ne 1/r_{2_{loss}}$ (dotted lines), starting with System 1 (red) or starting with System 2 (blue) or starting with any system with probability 0.5 (green)

Preferences in System 2 are assumed to be constructed by expected utility theory. The expected utility of an option is the utility of the outcome *u*(*x*), times the probability of its occurrence. For simplicity, I assume a risk-neutral DM, i.e., $u(x) = x$. As shown above, it does not affect any of the qualitative predictions. The expected utility, therefore, equals the expected value (EV), here

22 $$\begin{aligned} EU(x, p) = EV(p,x) = p \cdot u(x) = p\cdot x. \end{aligned}$$

Operating in System 2 produces a mean difference in valence related to EU values,

23 $$\begin{aligned} \mu _{2} = EV(G) -EV(S), \end{aligned}$$

independent of a specific frame. Because the expected value for the gamble and the sure option was identical, the drift rate is set to 0 (indifference between the game and the sure option).

The criteria $\theta _{TP}$ and $\theta _{noTP}$ for time pressure and no time pressure conditions, respectively, were symmetric, i.e., no a priori bias was assumed towards either choice option. The non-decision, residual times for each deadline condition were $R_{TP}$ and $R_{noTP}$. These eight parameters $\alpha , \beta , \gamma , \lambda , \theta _{TP}, \theta _{noTP}, R_{TP}$, and $R_{noTP}$ apply to both model architectures.

The two-stage dual process model (serial processing) assumes that the System 1 is followed by System 2. Assuming a geometric distribution for operating in System 1 before switching to System 2, the mean times were $E(T_{gain})=1/r_{gain}$ and $E(T_{loss})=1/r_{loss}$, respectively for the gain and the loss frame. They were assumed to be the same for the two different deadlines. In total, there are 10 model parameters to be estimated from the data.

For the quasi-parallel dual process model the order schedule needs to be specified. Either System 1 comes first, or System 2 comes first, or both systems have a specified probability of operating first. This adds one more model parameter. Furthermore, the operating times of the two systems may be different, adding four more parameters: $1/r_{1_{gain}}$, $1/r_{1_{loss}}$, $1/r_{2_{gain}}$, and $1/r_{2_{loss}}$. In total, there are 13 model parameters to be estimated from the data.

Here, I first compare the accounts of the serial model and the “true” parallel model (same operation distributions and means for both systems and fixing the probability to 0.5 for starting with any system), and then I compare six parallel versions (Fig. 11).

Figure 12 shows the observed choice frequencies (first row) and mean choice response times (second and third row) for the condition no time constraints (noTP) (left two columns) and the condition time constraints (TP) (right two columns), each performed under a gain or a loss frame and the model accounts of the serial model version (red lines ) and the parallel model version (black dotted lines). Both model versions (serial model and the “true’’ parallel) can account for the choice frequencies observed in the gain frame. For the loss frame, only the serial model version can account for them. Both model versions have problems to account for the entire patterns of observed mean choice response times. One reason might be that the data are not monotonically increasing or decreasing as a function of the amount given. Going back to Eqs. (18), (19), (22) shows that monotonicity would be expected. Another reason might be the parsimonious assumptions of parameters. For instance, the residual, non-decision time is assumed to be the same for both frames. Thus, times seem over/underestimated to account for the diverse patterns. Note, however, that the framework is not meant as a data-fitting tool (measurement model) but rather as cognitive model with clear predictions about patterns. Assuming a (ad hoc) distribution on residual times or distributions accounting for individual differences (specifying (ad hoc) prior distributions) may improve the numerical fits but is not intended in the current paper.

Next, I compare the accounts of six parallel model versions shown in Fig. 13. As before, the observed choice frequencies are in the first row; the mean choice response times in the second and third row for the condition no time constraints (noTP) (left two columns) and the condition time constraints (TP) (right two columns), each performed under a gain or a loss frame. The model versions assume $1/r_{1_{gain}} = 1/r_{2_{gain}}$ and $1/r_{1_{loss}} =1/r_{2_{loss}}$ (Fig. 13, solid lines) or $1/r_{1_{gain}} \ne 1/r_{2_{gain}}$ and $1/r_{1_{loss}} \ne 1/r_{2_{loss}}$ (dotted lines), starting with System 1 (red) or starting with System 2 (blue) or starting with any system with probability 0.5 (green). Whereas the predicted choice probabilities under a gain frame are very similar for both time constraint conditions, the predicted choice probabilities under a loss frame are substantially different. The predicted choice response time patterns also depend on the model versions.

Table 1 shows the AIC of all models accounts. Because the order schedule was fixed here, it did not count as an additional parameter. As can seen, the AIC for the serial version and the parallel version, starting with System 1 and allowing the mean operating time in both systems to be different are similar. This is easy to explain because the estimated $1/r_2$ can be adjusted (small or large) to mimic the serial model version.

## Digression: More than Two Sub-processes

The dual-process approach obviously includes only two processes or systems. By a reviewer’s request, I briefly elaborate on extending the framework to more than two sub-processes (Diederich, 1997; Diederich & Oswald, 2014). Note that in previous applications the multistage approach has often been linked to experimental manipulations, for instance, to the number of attributes, the duration of a stimulus being presented, stimulus onset asynchronies, and so on. In those cases, the number of subprocesses was known and often the order in which they may occur as well. In all cases, a serial processing approach was assumed.

Here we assume finitely many underlying sub-processes in which the decision maker operates one-by-one for a certain period of time in some order and possibly with repetition. Different situations need to be distinguished. The order in which sub-processes are tapped on may be known (e.g., by some evidence from neuroscience). The time each sub-process is operating may also be known (e.g., by physiological measures and imaging techniques). However, it is possible that neither the order in which sub-processes are operating nor the time spent operating in it are known.

Earlier I defined the specific order in which sub-processes are operating as *order schedule* and the times at which operating in one process switches to another one as *time schedule*. Both are part of the model with specific parameters. The order and time schedules may be given deterministically or randomly or a combination of it. When order and time schedules are deterministic, the schedule is called * deterministic time and order schedule*. When both schedules are random, i.e., when neither the order in which sub-processes operate in nor the operating time are known, it is called *random time and order schedule*. When one of the schedules is random and the other is deterministic, it is called a *semi-random schedule*.

**Table 1:** AIC for the 7 fitted model versions (serial (S) and 6 parallel (P))

Model	start with System	*E*(*T*)	#Parameters	AIC
S	1	$1/r_1$	10	814
P	1	$1/r_1=1/r_2$	10	18510
P	2	$1/r_1=1/r_2$	10	3348
P	any	$1/r_1=1/r_2$	10	8944
P	1	$1/r_1 \ne 1/r_2$	12	822
P	2	$1/r_1 \ne 1/r_2$	12	2195
P	any	$1/r_1 \ne 1/r_2$	12	1320

### Time Schedule

The time when switching from operating in one sub-process to operating in another sub-process occurs in a sequence and is formally defined as

24 $$\begin{aligned} {T}_0=T_{start}=0< { T}_1< { T}_2< \ldots < { T}_L={ T}_{end}, \end{aligned}$$

with ${T}_{end}$ representing the maximum duration of the entire decision process, called *time horizon*. On a theoretical level, it is possible to assume ${T}_{end}=\infty$ (infinite time horizon) and $L=\infty$ (infinite switching). $\Delta {T}_l= ({T}_{l-1},{T}_l]$ is the *l*-th *operating time interval*. That is, the time the decision maker operates in process *l*. Note that *T* may be a constant (deterministic time schedule) or a random variable (random time schedule). See Diederich & Oswald (2014) for several distribution examples.

### Order Schedule

For generating the order $\{k_l\}_{l=1,\ldots ,L}$ in which sub-processes operate, consider stochastic $K\times K$ matrices $D^{(l)}$ such that $d^{(l)}_{k'k}\ge 0$ describes the probability with which operating switches from the $k'$-th sub-process to the *k*-th sub-process at switching time $t_l$, $l=1,\ldots ,L-1$. Setting $d^{(l)}_{kk}=0$ avoids a no switching situation.

For example, assuming two sub-processes as in the dual process approach $$K=2$$, and $$d^{(l)}_{11}=d^{(l)}_{22}=0$$, $$d^{(l)}_{12}=d^{(l)}_{21}= $$ $$ 1$$, the sub-process sequence is either $$(1,2,1,2,\ldots )$$ or $$(2,1,2,1,\ldots )$$, depending on whether $$k_1=1$$ or $$k_1=2$$. For three sub-process and $$L=3$$, and setting

$$ D^{(1)} = \left[ \begin{array}{ccc} 0 &{} d_{12}^{(1)} &{} d_{13}^{(1)}\\ d_{21}^{(1)} &{} 0 &{} d_{23}^{(1)}\\ d_{31} &{} d_{32}^{(1)} &{} 0\end{array} \right] ,\qquad D^{(2)} = \left[ \begin{array}{ccc} 0 &{} 1 &{} 0\\ 1 &{} 0 &{} 0\\ d_{31}^{(2)} &{} d_{32}^{(2)} &{} 0\end{array} \right] , $$

and $k_1=1$ result in order sequences (1, 2, 1), (1, 3, 1), (1, 3, 2) with probability $d_{12}^{(1)}$, $d_{13}^{(1)}\cdot d_{31}^{(2)}$, $d_{13}^{(1)}\cdot d_{32}^{(2)}$, respectively.

A time and order schedule consists of a sequence $\{{T}_l\}_{l=1,\ldots ,}$ ${L}$ of operating switching times, and a sequence $\{k_{l} \in \{1,\ldots ,\kappa \}\}_{l=1,\ldots ,L}$ of sub-process indices which specifies that during the time interval $\Delta {T}_l$ the $k_l$-th sub-process is operating. At switching time ${T}_l$, $l=1,\ldots ,L-1$, the decision process switches from operating in sub-process $k_l$ to operating in sub-process $k_{l+1}$. As before, the entire process *X*(*t*) determined by such a schedule is a *piecewise* diffusion process with fixed parameters in each interval $\Delta {T}_l$.

For derivations and examples see Diederich (1997); Diederich & Oswald (2014, 2016); Diederich & Mallahi-Karai (2018).

## Concluding Remarks

Over the last five decades, the idea of dual processes has been very influential in psychology and increasingly so in behavioral economics and neuroscience. The two systems are often assumed to operate in an antagonistic manner like fast versus slow or intuitive versus rational. With a few exceptions (e.g., Brocas & Carrillo, 2014; Fudenberg & Levine, 2006; Klauer et al., 2010; Loewenstein et al., 2015; Mukherjee, 2010), though none of them dynamic, dual process models are only verbal descriptions of the systems and their interaction is not made explicit. Attribution to one system or the other is often made ad hoc and in hindsight. Often fast answers are linked to System 1 and slow answers are linked to System 2. As Krajbich et al. (2015) discussed in detail, such reverse inference is problematic because it does not take into account other sources of variability in the data that can influence choice speed (e.g., choice difficulty). The current framework specifies the dynamics, interactions, and, in particular, the architecture of the two systems, i.e., sequential versus running in parallel. Comparing the predictions of both architectures shows the following. For the serial process, the magnitudes of the mean valences (drift rates) in both systems matter: If $\mu _1> \mu _2 > 0$ the model predicts shorter response times for the more frequently chosen alternative. If $0< \mu _1 < \mu _2$ the model predicts faster responses for the less frequently chosen alternative. For $\mu _1$ and $\mu _2$ pointing in opposite directions a preference reversal occurs with increasing operation times in System 1. For parallel processing, the system with the larger mean valence (in absolute terms) determines the overall direction of preferences. No crossover of choice probabilities occurs. Furthermore, the mean choice response times for choosing the sure option and the gamble are very similar. Seven model versions have been tested on published data (Guo et al., 2017). For those data, the two-stage dual process model (serial architecture) gave the best accounts closely followed by parallel version assuming that the entire process starts with System 1 and then switches back and for the between the two systems with different operation times each system. Importantly, while some of the models gave similar accounts for some of the choice frequencies they differed substantially with respect to the predicted choice response patterns. Collecting times is therefore advisable. Thus, formalizing the dual process assumptions to allow for quantitative and qualitative predictions and rigorous testing will solve some of the issues that proposers and opponents of this approach hold.

The framework can be extended to more complex decision situations including more than two choice alternatives. In recent work we proposed three models that account for multiple choice alternatives: The 2N-ary choice tree model (Wollschläger & Diederich, 2012, 2017, 2020) (a random walk on a tree with positive and negative counters), the cube model (Mallahi-Karai & Diederich, 2019) and the disk model (Mallahi-Karai & Diederich, 2021), both geometric models in higher dispersions with accept and reject boundaries. As for other dynamic-stochastic models with multiple alternatives and attributes (e.g., Roe et al., 2001; Trueblood et al., 2014; Usher & McClelland, 2004) all information about the attributes of one choice alternative is mapped onto a single drift rate. The attribute information could be modeled with the approach described in Section 5. We are working on the feasibility of including these assumptions, which is not trivial at all. Note, however, that the assumed antagonistic characteristics of the dual process approach mainly asks for binary designs and extended it to more than two choice alternatives may be questionable and is certainly beyond the scope of the present paper.

## Figures and Tables

**Fig. 14:**
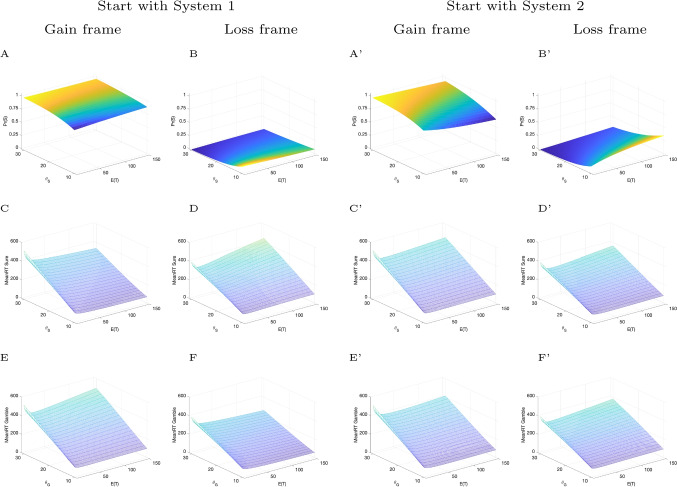
Parallel dynamic-stochastic dual process model either starting with System 1 (left two columns) or starting with System 2. Risk-neutral DM. Predicted choice probabilities for choosing the sure option in a gain frame (**A** and **A’**) and a loss frame (**B** and **E’**). Predicted mean choice response times for choosing the sure option in a gain frame (**C** and **C’**) and a loss frame (**D** and **D’**). Predicted mean choice response times for choosing the gamble in a gain frame (**E** and **E’**) and a loss frame (**F** and **F’**)

**Fig. 15:**
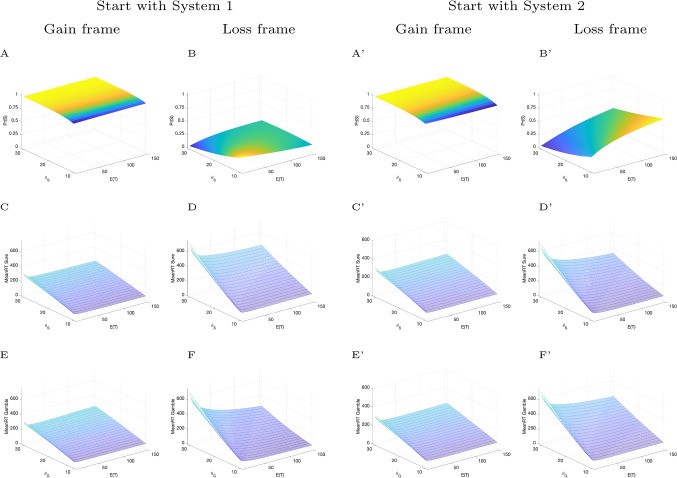
Parallel dynamic-stochastic dual process model either starting with System 1 (left two columns) or starting with System 2. Risk-avoiding DM. Predicted choice probabilities for choosing the sure option in a gain frame (**A** and **A’**) and a loss frame (**B** and **E’**). Predicted mean choice response times for choosing the sure option in a gain frame (**C** and **C’**) and a loss frame (**D** and **D’**). Predicted mean choice response times for choosing the gamble in a gain frame (**E** and **E’**) and a loss frame (**F** and **F’**)

**Fig. 16:**
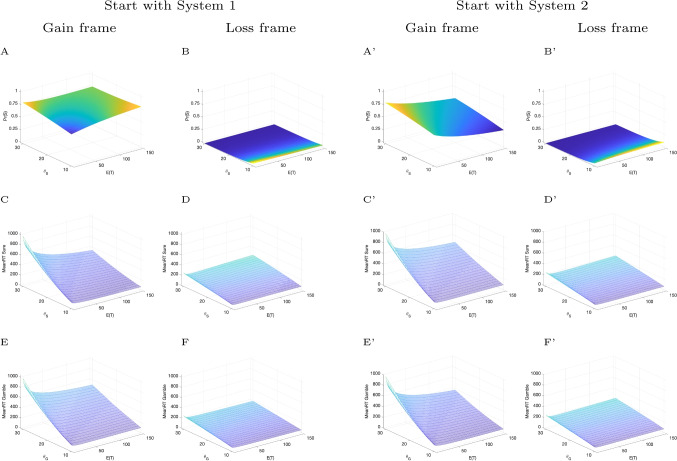
Parallel dynamic-stochastic dual process model either starting with System 1 (left two columns) or starting with System 2. Risk-seeking DM. Predicted choice probabilities for choosing the sure option in a gain frame (**A** and **A’**) and a loss frame (**B** and **E’**). Predicted mean choice response times for choosing the sure option in a gain frame (**C** and **C’**) and a loss frame (**D** and **D’**). Predicted mean choice response times for choosing the gamble in a gain frame (**E** and **E’**) and a loss frame (**F** and **F’**)

## Appendix A

Figures 14, 15, and 16 show the predicted choice probabilities and mean choice response times when the process starts operating in any system (left two columns) or when the process starts operating in System 1.

## Footnotes

1 In the following I will use the generic terms System1 and System 2 throughout. Following (DeNeys, 2021) I will use the terms “process” and “system” interchangeably as well as theory, model, and framework in this context.

2 The question of how to distinguish parallel versus serial processing has a long history in mathematical psychology, initiated by the classic paper of Townsend (1972). Asserting the (non-)equivalence of parallel and serial systems requires additional formal definitions going beyond the realm of this paper (see Townsend, 1990). Furthermore, it requires experimental manipulations that are not feasible in the current context.

3 Technically, ${{V}}(t)= \mu t + \sigma W(t)$ is a Wiener process with drift $\mu$, where *W*(*t*) is the standard Wiener process with Gaussian increments. For simplicity, the diffusion coefficient is set to 1. An alternative process is an Ornstein-Uhlenbeck Process (OUP), not discussed here (see, e.g., Busemeyer & Townsend, 1993; Diederich, 1997).

4 Note that time pressure may be incorporated into the model by assuming different drift rates and/or different operating times in System 1. The drift rate assumption is rather ad hoc and needs justification. The extended operating time in System 1 is a testable assumption.

5 The basic assumption of race models is that two racers are presented by two random variables, say X and Y, and the minimum of them, i.e., min(X,Y) determines the response. However, most of the dual process models assume that System 1 (presented by X) is always faster than System 2 (presented by Y). That is, if the probability of X being smaller than Y is 1 ($Prob(X < Y) = 1$) this is equivalent to *X* being stochastically dominated by *Y*, $F_X(t) \ge F_Y(t)$ for all *t*.

6 That means, each system has a probability of 0.5 to operate first. Obviously, it is possible to assume that one system starts with probability *p* and the other with one and $1-p$. It can also be generalized by assuming a probability distribution over starting values.

7 When estimating the parameters from the data, however, the order is reflected in an additional model parameter.
